# A variant within the *FTO* confers susceptibility to diabetic nephropathy in Japanese patients with type 2 diabetes

**DOI:** 10.1371/journal.pone.0208654

**Published:** 2018-12-19

**Authors:** Makiko Taira, Minako Imamura, Atsushi Takahashi, Yoichiro Kamatani, Toshimasa Yamauchi, Shin-ichi Araki, Nobue Tanaka, Natalie R. van Zuydam, Emma Ahlqvist, Masao Toyoda, Tomoya Umezono, Koichi Kawai, Masahito Imanishi, Hirotaka Watada, Daisuke Suzuki, Hiroshi Maegawa, Tetsuya Babazono, Kohei Kaku, Ryuzo Kawamori, The SUMMIT Consortium, Leif C. Groop, Mark I. McCarthy, Takashi Kadowaki, Shiro Maeda

**Affiliations:** 1 Laboratory for Endocrinology, Metabolism and Kidney Diseases, RIKEN Center for Integrative Medical Sciences, Kanagawa, Japan; 2 Department of Advanced Genomic and Laboratory Medicine, Graduate School of Medicine, University of the Ryukyus, Okinawa, Japan; 3 Division of Clinical Laboratory and Blood Transfusion, University of the Ryukyus Hospital, Okinawa, Japan; 4 Laboratory for Statistical Analysis, RIKEN Center for Integrative Medical Sciences, Kanagawa, Japan; 5 Department of Genomic Medicine, Research Institute, National Cerebral and Cardiovascular Center, Osaka, Japan; 6 Kyoto-McGill International Collaborative School in Genomic Medicine, Kyoto University Graduate School of Medicine, Kyoto, Japan; 7 Department of Diabetes and Metabolic Diseases, Graduate School of Medicine, The University of Tokyo, Tokyo, Japan; 8 Department of Medicine, Shiga University of Medical Science, Shiga, Japan; 9 Diabetes Center, Tokyo Women’s Medical University School of Medicine, Tokyo, Japan; 10 Wellcome Centre for Human Genetics, Nuffield Department of Medicine, University of Oxford, Oxford, United Kingdom; 11 Oxford Center for Diabetes, Endocrinology and Metabolism, Radcliffe Department of Medicine, University of Oxford, Oxford, United Kingdom; 12 Department of Clinical Sciences, Diabetes and Endocrinology, Lund University Diabetes Centre, Scania University Hospital, Malmö, Sweden; 13 Division of Nephrology, Endocrinology and Metabolism, Department of Internal Medicine, Tokai University School of Medicine, Kanagawa, Japan; 14 Kawai Clinic, Ibaragi, Japan; 15 Division of Nephrology and Hypertension, Department of Internal Medicine, Osaka City General Hospital, Osaka, Japan; 16 Department of Nephrology, and Hemodialysis Unit, Ishikiriseiki Hospital, Higashi-Osaka, Japan; 17 Department of Metabolism and Endocrinology, Juntendo University Graduate School of Medicine, Tokyo, Japan; 18 Sportology Center, Juntendo University Graduate School of Medicine, Tokyo, Japan; 19 Suzuki Diabetes Clinic, Kanagawa, Japan; 20 Department of Internal Medicine, Kawasaki Medical School, Okayama, Japan; 21 Institute for Molecular Medicine Finland, University of Helsinki, Helsinki, Finland; 22 Oxford NIHR BIOMEDICAL Research Centre, Oxford University Hospitals Trust, Oxford, United Kingdom; 23 Department of Prevention of Diabetes and Lifestyle-Related Diseases, Graduate School of Medicine, The University of Tokyo, Tokyo, Japan; 24 Department of Metabolism and Nutrition, Mizonokuchi Hospital, Faculty of Medicine, Teikyo University, Kanagawa, Japan; Kunming Institute of Zoology, Chinese Academy of Sciences, CHINA

## Abstract

To explore novel genetic loci for diabetic nephropathy, we performed genome-wide association studies (GWAS) for diabetic nephropathy in Japanese patients with type 2 diabetes. We analyzed the association of 5,768,242 single nucleotide polymorphisms (SNPs) in Japanese patients with type 2 diabetes, 2,380 nephropathy cases and 5,234 controls. We further performed GWAS for diabetic nephropathy using independent Japanese patients with type 2 diabetes, 429 cases and 358 controls and the results of these two GWAS were combined with an inverse variance meta-analysis (stage-1), followed by a *de novo* genotyping for the candidate SNP loci (p < 1.0 × 10^−4^) in an independent case-control study (Stage-2; 1,213 cases and 1,298 controls). After integrating stage-1 and stage-2 data, we identified one SNP locus, significantly associated with diabetic nephropathy; rs56094641 in *FTO*, *P* = 7.74 × 10^−10^. We further examined the association of rs56094641 with diabetic nephropathy in independent Japanese patients with type 2 diabetes (902 cases and 1,221 controls), and found that the association of this locus with diabetic nephropathy remained significant after integrating all association data (*P* = 7.62 × 10^−10^). We have identified *FTO* locus as a novel locus for conferring susceptibility to diabetic nephropathy in Japanese patients with type 2 diabetes.

## Introduction

Diabetic nephropathy is a leading cause of end-stage renal disease (ESRD), and its prevalence is progressively increasing according to the increase of number of patients with diabetes mellitus [1,2]. Prolonged persistent hyperglycemia is a principal cause of diabetic microvascular complications, but it has been shown that only ~30% of all patients with diabetes develop overt nephropathy, whereas most patients develop diabetic retinopathy 20–30 years after the onset of diabetes [3]. In addition, familial clustering of diabetic nephropathy has been observed in both type 1 and type 2 diabetes [4–6], implying that genetic factors are involved in the development and/or progression of diabetic nephropathy. Identification of disease susceptibility loci for many common diseases such as type 2 diabetes, has been achieved by the introduction of genome-wide association studies (GWAS). Worldwide efforts to identify susceptibility loci for diabetic nephropathy, however, have not yet met with success so much. Several susceptibility loci to diabetic nephropathy or its related traits which showed their lowest p-values close to or with a genome-wide significance level have been unveiled; rs2268388 in *ACACB* [7], rs7583877 in *AFF3* locus, rs17709344 in *RGMA-MCTP2* locus [8], rs4972593 in *Sp3-CDCA7* locus [9], rs1801239 in *CUBN* locus [10], rs161740 in *EPO* locus [11]. However, the results have not been conclusive, and thus most susceptibility loci for diabetic nephropathy remain to be un-identified, suggesting heterogeneity of the disease or contribution of non-genetic factors, which have not been taken into account, may produce inconsistent results among the studies.

In this study, to identify novel susceptibility loci to diabetic nephropathy, we performed a GWAS meta-analysis for diabetic nephropathy using existing Japanese GWAS data for patients with type 2 diabetes.

## Materials and methods

### Study subjects

#### Discovery stage (Stage-1)

We selected 7,641 individuals having type 2 diabetes registered in Biobank Japan, and divided these patients into two groups (stage-1, set-1); 1) 2,380 nephropathy cases, defined as patients with overt albuminuria or under renal replacement therapy and 2) 5,234 controls with normoalbuminuria and with diabetes duration of 5 years or longer or with diabetic retinopathy. We also used independent patients with type 2 diabetes who were extracted from previously reported GWAS data [12], and performed GWAS for diabetic nephropathy (stage-1 set 2, cases, n = 429, controls, n = 358). There was no overlap between set-1 and set-2. Diabetes was diagnosed according to the World Health Organization (WHO) criteria [13], and those who were diagnosed as type 1 diabetes, mitochondrial diseases or maturity-onset diabetes of the young were excluded.

#### Validation analysis (Stage-2)

We examined independent 2,511 patients with Japanese patients with type 2 diabetes, 1,213 diabetic nephropathy cases and 1,298 controls, from the BioBank Japan that were not included in the discovery stage.

Clinical characteristics of participants for Stage-1 (set-1, set-2) and Stage-2 are shown in Table 1. Genomic DNA was extracted from peripheral leukocytes using the standard procedure. All individuals provided written informed consent to participate in this study.

**Table 1:** Clinical characteristics of participants.

	Stage1				Stage2	
	Set1		Set2			
	case	control	case	control	case	control
n	2,380	5,234	429	358	1,213	1,298
M/F	1,611/769	3,162/2,072	304/125	226/131**	833/380	759/539
Men%	67.6	60.4	70.9	63.1	68.6	58.5
age	65.9 ± 10.5	66.3 ± 9.6	67.2 ± 10.0	66.9 ± 9.2	63.9 ± 11.0	63.9 ± 10.5
BMI	23.9 ± 4.0*	23.6 ± 3.6	23.5 ± 4.0	23.8 ± 3.8	24.1 ± 3.9	23.6 ± 3.8
Diabetes duration	13.1 ± 9.8*	14.4 ± 8.5	13.4 ± 10.2	13.2 ± 10.0	12.0 ± 9.5	10.5 ± 8.8

* P < 0.01 vs. control

** information of sex for one participant is not available

### Genotyping, quality control and imputation in the discovery stage

Set-1 individuals in stage 1 were genotyped using the Human Omni Express Exome Bead Chip. There were 628,670 autosomal SNPs that passed quality control, a call rate ≥ 0.99, Hardy-Weinberg equilibrium test P ≥ 1 × 10^−6^ in controls and minor allele frequency (MAF) ≥ 0.01. Set-2 samples were genotyped using the Illumina Human 610K SNP array, and 504,984 autosomal SNPs passed the quality control described above and used for further analysis. For sample quality control, we evaluated cryptic relatedness for each sample using an identity-by-state method and removed samples that exhibited second-degree or closer relatedness. We performed principal component analysis to select individuals belong to the major Japanese cluster (Hondo cluster, S1 Fig) as reported previously [14], and data for 7,614 individuals (2,380 diabetic nephropathy cases and 5,234 controls) in set-1 and 787 individuals (429 diabetic nephropathy cases and 358 controls) in set-2 were used in subsequent analyses. We performed genotype imputation with MACH and Minimac [15,16] using linkage disequilibrium data in the 1000 Genomes Project (phased JPT, CHB and Han Chinese South data n = 275, March 2012) as reference populations. To evaluate the potential effect of population stratification, we used a quantile-quantile (qq) plot of the observed P-values.

### *De novo* genotyping

We genotyped 1,213 individuals with diabetic nephropathy and 1,298 controls (Stage-2) registered as type 2 diabetes in BioBank Japan, who were not included in a discovery stage using a multiplex PCR-invader assay [17] for SNPs with p values < 10^−4^ in the meta-analysis. We also genotyped additional 2,123 Japanese patients with type 2 diabetes (902 diabetic nephropathy cases and 1,221 controls), who visited out-patient clinics of Tokai university hospital, Shiga university of medical science hospital, Juntendo university hospital, Kawasaki medical school hospital, Tokyo women’s medical university hospital, Iwate Medical University, Toride Kyodo Hospital, Kawai Clinic, Osaka City General Hospital, Chiba Tokusyukai Hospital or Osaka Rosai Hospital.

Genotyping success rates < 95% or concordance rates < 99.9% were excluded from the analyses.

### Statistical analysis

The association between each SNP and diabetic nephropathy was assessed using the logistic regression test with an additive model with or without adjusting for age, sex, and log-transformed body mass index (BMI) using Mach2dat. We combined data from the each GWAS, validation and replication studies using METAL [18] as an inverse variance method. Heterogeneity in effect sizes among the studies was evaluated with a Cochran’s Q test. Regional association plots were generated using LocusZoom [19].

### Ethics approval

The protocol of this study conformed to the provisions of the Declaration of Helsinki and was approved by the ethical committees at the RIKEN Yokohama Institute, Tokai university hospital, Shiga university of medical science, Juntendo university, Kawasaki medical school, Tokyo women’s medical university and Iwate Medical University, and the institutional review boards at Toride Kyodo Hospital, Osaka City General Hospital, Chiba Tokusyukai Hospital and Osaka Rosai Hospital.

## Results

### A meta-analysis of GWAS for diabetic nephropathy in the Japanese patients with type 2 diabetes

**Fig 1:**
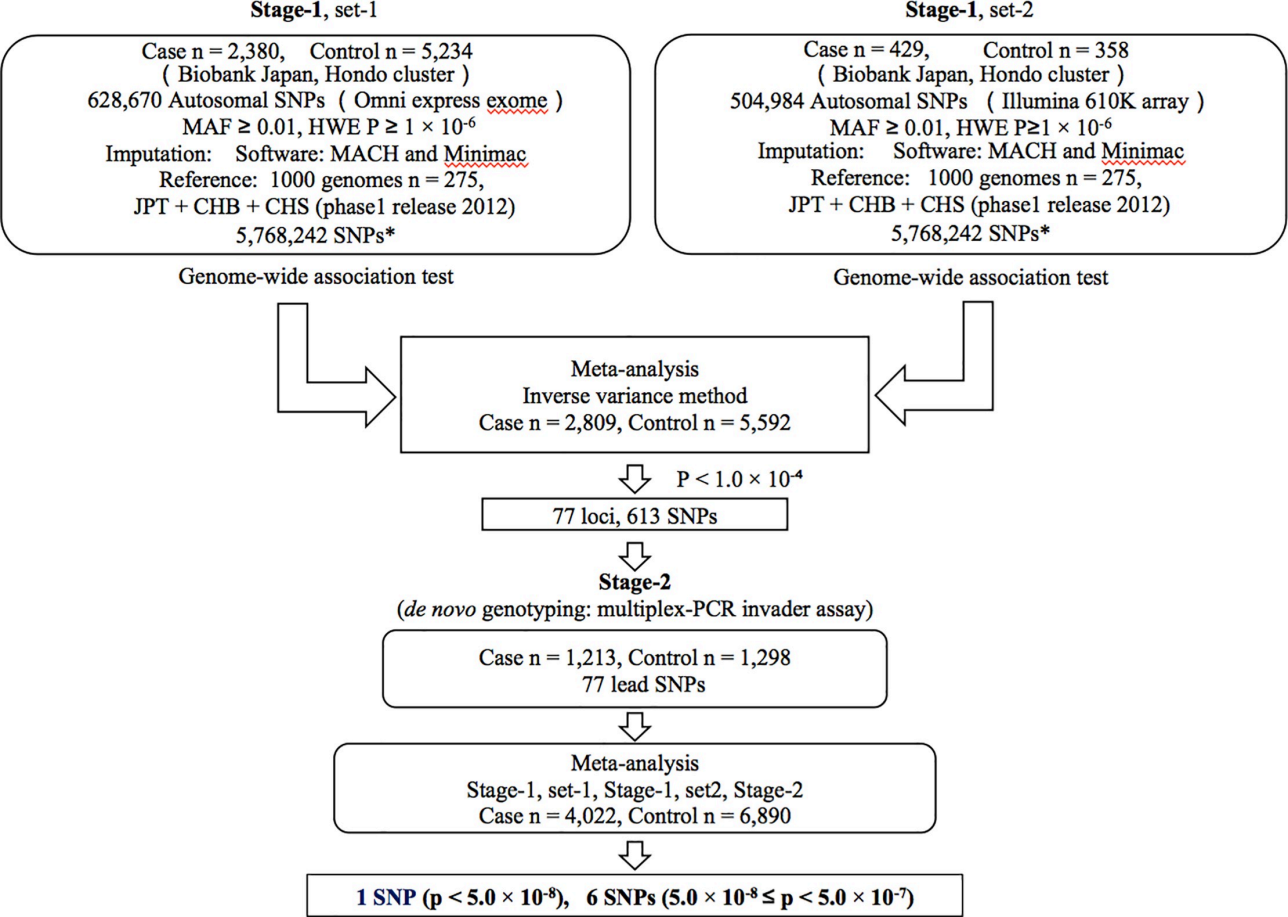
Outline of this study. * r^2^ > 0.7 in both studies, SNP; single nucleotide polymorphism, MAF; minor allele frequency, HWE P; Hardy-Weinberg Equilibrium test P, RSQ, r square.

We obtained genotype data for 7,521,074 SNPs by imputation, and among them, 5,768,242 SNPs those passed quality control (r^2^ > 0.7) in both studies (Stage-1 set-1 and set-2) were evaluated in this meta-analysis (Fig 1).

There was no genomic inflation in the qq plots in the both studies (S2 Fig). We selected 77 loci associated with diabetic nephropathy in the meta-analysis (*P* < 1 × 10^−4^, S1 Table), and the associations of the lead SNPs from the 77 loci with DN were examined in an independent case-control study (Stage-2, 1,213 cases and 1,298 controls). In stage-2 analyses, we successfully obtained the genotype data for 65 loci by the multiplex-PCR invader assay. After combining all association data (Stage-1 set-1, set-2 and Stage-2) using the inverse-variance fixed-effects meta-analysis, we found that one SNP locus, rs56094641 in *FTO* at chromosome (Chr) 16, 16q12.2, showed a genome-wide significant association with diabetic nephropathy (*P* = 7.74 × 10^−10^, odds ratio (OR) = 1.23, 95% confidence interval (CI) 1.15−1.31, Table 2 and S2 Table). The association of the *FTO* locus with overt nephropathy was not affected by an adjustment for age, sex and BMI (rs9936385, r^2^ = 1 with rs56094641, S3 Table). We additionally identified suggestive evidence for the associations of six SNP loci with diabetic nephropathy (5.0 × 10^−8^ < P < 5.0 × 10^−7^, Table 2 and S2 Table): rs895157 in *PRCD* (17q25.1, *P* = 7.70 × 10^−8^, OR = 1.28, 95% CI 1.17−1.41), rs10144968 near *RAD51B* (14q24.1–24.2, *P* = 1.22 × 10^−7^, OR = 1.35, 95% CI 1.21−1.51), rs13306536 in *LRP8* (1p32.3, *P* = 2.70 × 10^−7^, OR = 1.33, 95% CI 1.19−1.48), rs7544082 near *TRABD2B* (1p33, *P* = 3.08 × 10^−7^, OR = 1.17, 95% CI 1.10−1.24), rs11101179 in *CHAT* (10q11.2–21.1, *P* = 3.85 × 10^−7^, OR = 1.19, 95% CI 1.11−1.28), rs710375 near *CCNH* (5q14-15, *P* = 3.96 × 10^−7^, OR = 1.21, 95% CI 1.12−1.30). Regional plots for these 7 loci are shown in S3 Fig.

**Table 2:** Association of 7 SNP loci with diabetic nephropathy in Japanese patients with type 2 diabetes.

SNP ID Gene Chromosome	Alleles	study	RAF	OR (95%CI)	P	Phet
rs56094641	G/A	Stage-1, set-1	0.247/0.211	1.22 (1.13–1.32)	1.19 × 10^−6^	
*FTO*		Stage-1, set-2	0.236/0.207	1.19 (0.93–1.52)	0.164	
Ch16		Stage-2	0.252/0.209	1.27 (1.11–1.46)	3.76 × 10^−4^	
		**Combined**		**1.23 (1.15–1.31)**	**7.74 × 10**^**−10**^	0.85
rs895157	C/A	Stage-1, set-1	0.130/0.108	1.28 (1.14–1.43)	2.85 × 10^−5^	
*PRCD*		Stage-1, set-2	0.126/0.113	1.14 (0.83–1.58)	0.422	
Ch17		Stage-2	0.140/0.109	1.35 (1.13–1.60)	7.02 × 10^−4^	
		**Combined**		**1.28 (1.17–1.41)**	**7.70 × 10**^**−8**^	0.67
rs10144968	G/T	Stage-1, set-1	0.078/0.062	1.27 (1.11–1.46)	3.75 × 10^−4^	
*RAD51B*		Stage-1, set-2	0.083/0.060	1.41 (0.95–2.10)	8.48 × 10^−2^	
		Stage-2	0.078/0.051	1.58 (1.25–1.99)	1.15 × 10^−4^	
		**Combined**		**1.35 (1.21–1.51)**	**1.22 × 10**^**−7**^	0.28
rs13306536	T/C	Stage-1, set-1	0.089/0.068	1.27 (1.11–1.46)	3.75 × 10^−4^	
*LRP8*		Stage-1, set-2	0.068/0.075	0.87 (0.58–1.30)	0.496	
Ch1		Stage-2	0.076/0.058	1.35 (1.08–1.69)	1.15 × 10^−4^	
		**Combined**		**1.33 (1.19–1.48)**	**2.70 × 10**^**−7**^	0.1
rs7544082	A/C	Stage-1, set-1	0.525/0.492	1.18 (1.09–1.27)	3.22 × 10^−5^	
*TRABD2B*		Stage-1, set-2	0.550/0.514	1.15 (0.77–1.72)	0.164	
Ch1		Stage-2	0.552/0.514	1.16 (1.04–1.31)	8.70 × 10^−3^	
		**Combined**		**1.17 (1.10–1.24)**	**3.08 × 10**^**−7**^	0.97
rs11101179	C/T	Stage-1, set-1	0.231/0.197	1.22 (1.12–1.33)	2.23 × 10^−6^	
*CHAT*		Stage-1, set-2	0.205/0.205	1.00 (0.77–1.31)	0.977	
Ch10		Stage-2	0.226/0.198	1.17 (1.02–1.34)	2.10 × 10^−2^	
		**Combined**		**1.19 (1.11–1.28)**	**3.85 × 10**^**−7**^	0.37
rs710375	C/T	Stage-1, set-1	0.201/0.169	1.24 (1.14–1.36)	2.23 × 10^−6^	
*CCNH-TMEM161B*		Stage-1, set-2	0.197/0.154	1.38 (1.04–1.77)	0.977	
Ch5		Stage-2	0.187/0.176	1.08 (0.93–1.24)	2.10 × 10^−2^	
		**Combined**		**1.21 (1.12–1.30)**	**3.96 × 10**^**−7**^	0.16

### Replication study

We examined the association of rs56094641 in *FTO* with overt nephropathy in 2,123 Japanese patients with type 2 diabetes (cases n = 902, controls n = 1,221). As shown in Table 3, rs56094641 in *FTO* showed the same direction of effect with the original finding in the discovery stage. The association of rs56094641 in *FTO* with overt nephropathy was still genome-wide significant level after integration of all association data (*P* = 7.62 × 10^−10^, OR = 1.21, 95% CI 1.14−1.29, Table 3) though the association was not statistically significant in the replication study alone (*P* = 0.258, OR = 1.11, 95% CI 0.93−1.32, Table 3).

By *in silico* replication using SUMMIT consortium data for type 2 diabetes, the association of *FTO* variants with diabetic nephropathy was not replicated in European patients with type 2 diabetes (Table 3).

**Table 3:** Replication studies for 7 loci associated with diabetic nephropathy.

SNP ID gene	Alleles	Ethnicity	RAF	OR (95%CI)	P	Phet
rs56094641	G/A	Japanese	Discovery	1.23 (1.15–1.31)	7.74 × 10^−10^	
*FTO*			Replication	1.11 (0.93–1.32)	0.258	
			Combined	1.21 (1.14–1.29)	7.62 × 10^−10^	0.67
		Europeans		1.06 (1.19–0.95)	0.300	
rs895157	C/A	Japanese	Discovery	1.28 (1.17–1.41)	7.70 × 10^−8^	
*PRCD*			Replication	0.96 (0.77–1.21)	0.748	
			Combined	1.23 (1.13–1.34)	1.15 × 10^−6^	0.11
		Europeans		0.92 (0.80–1.06)	0.195	
rs10144968	G/T	Japanese	Discovery	1.35 (1.21–1.51)	1.22 × 10^−7^	
*RAD51B*			Replication	1.04 (0.76–1.41)	0.808	
			Combined	1.31 (1.18–1.45)	4.23 × 10^−7^	0.17
		Europeans		0.91 (0.80–1.03)	0.289	
rs13306536	T/C	Japanese	Discovery	1.33 (1.19–1.48)	2.70 × 10^−7^	
*LRP8*			Replication	0.98 (0.72–1.34)	0.914	
			Combined	1.30 (1.16–1.42)	1.38 × 10^−6^	0.05
		Europeans		N/A	N/A	
rs7544082	A/C	Japanese	Discovery	1.17 (1.10–1.24)	3.08 × 10^−7^	
*TRABD2B*			Replication	0.99 (0.86–1.15)	0.946	
			Combined	1.16 (1.04–1.31)	2.58 × 10^−6^	0.24
		Europeans		N/A	N/A	
rs11101179	C/T	Japanese	Discovery	1.19 (1.11–1.28)	3.85 × 10^−7^	
*CHAT*			Replication	0.92 (0.77–1.10)	0.365	
			Combined	1.15 (1.08–1.23)	9.36 × 10^−6^	0.03
		Europeans		N/A	N/A	
rs710375	C/T	Japanese	Discovery	1.21 (1.12–1.30)	3.96 × 10^−7^	
*CCNH*			Replication	0.85 (0.70–1.03)	9.86 × 10^−2^	
			Combined	1.16 (1.08–1.24)	3.02 × 10^−5^	0.002
		Europeans		1.12 (0.999–1.12)	3.58 × 10^−2^	

N/A, data is not available

### Evaluation of previously reported loci

We reflected our results (meta-analysis of Stage-1 set-1 and set-2) on previously- reported susceptibility loci to overt nephropathy. There was no overlap between the above seven SNP loci and 21 loci from previously-reported GWAS for susceptibility to overt nephropathy (S4 Table). None of the SNPs in the original reports showed significant association with overt nephropathy in the discovery stage (*P* > 2.38 x 10^−3^ = 0.05/21). Regional lead SNPs within the *ELMO1*, *RGMA*-*MCTP2*, *ERBB4* and *SP3*-*CDCA7* in the discovery stage attained the threshold of the correction of multiple testing error.

## Discussion

From a result of GWAS meta-analysis for diabetic nephropathy followed by validation studies in Japanese patients with type 2 diabetes, which comprising in 4,022 cases and 6,890 controls, we identified significant association between rs56094641 in *FTO* and susceptibility to diabetic nephropathy in Japanese patients with type 2 diabetes.

*FTO* locus has been repeatedly reported to be associated with obesity and/or adiposity [20], and, in this study, we have shown that the risk allele for obesity was significantly associated with susceptibility to diabetic nephropathy in Japanese patients with type 2 diabetes.

Smemo *et al*. mentioned that obesity-associated noncoding sequences within *FTO* are functionally connected, at megabase distances, with the homeobox gene *IRX3* [21]. *IRX3* is a member of the Iroquois homeobox gene family and plays a role in an early step of neural development [22]. Members of this family including *IRX3* and *IRX5* appear to play multiple roles during pattern formation of vertebrate embryos [23]. However, there is no evidence showing functional link between *IRX3*/*IRX5* and diabetic nephropathy.

Obesity has been recognized as an important risk factor for chronic kidney disease (CKD) [24,25]. The incidence of obesity has actualized an increase the number of patients with obesity-related glomerulopathy and proteinuria [26]. Furthermore, obesity has been reported to be an independent risk factor for ESRD by a large historical cohort study of 177,570 adults after adjustment for multiple epidemiologic and clinical conditions including diabetes [27] Moreover, a *FTO* variant, rs17817449, which are in absolute linkage disequilibrium to rs56094641, was shown to be associated with ESRD [28], suggesting *FTO* variants confer susceptibility to CKD/ESRD through the mechanisms mediated by obesity/adipocity.

In European patients with type 1 or type 2 diabetes, a genetic risk score constructed from confirmed obesity related SNP loci was associated with diabetic nephropathy [29–31], although *FTO* variant alone did not have significant effect on any of renal phenotype. Therefore, our report is the first to show a robust association of *FTO* variant with diabetic nephropathy. In addition, adjustment for BMI did not influence the association of the SNP with diabetic nephropathy in this study, suggesting that the SNP gets involved in susceptibility to diabetic nephropathy through the mechanism other than increasing BMI. Values of BMI used in this study, however, were obtained after making diagnosis of type 2 diabetes and some therapeutic interventions might affect the BMI values used in this study. Therefore, we might underestimate the effects of BMI on susceptibility to diabetic nephropathy.

We also obtained six SNP loci, which have shown borderline associations with overt nephropathy at the discovery stage (Table 1: 5 × 10^−8^ < combined *P* ≤ 5 × 10^−7^), but all candidate SNPs did not show significant effects in the replication stage in an independent Japanese case-control study (Table 3). In this study, we could not replicate almost all of variants identified by meta-analysis of stage 1 and stage 2 in the additional dataset with a comparable sample size with stage 2 GWAS. Although genome-wide SNPs data are not available for individuals in the replication stage, all samples for the replication stage were collected in the Japan main-island, and it has been shown individuals living in Japan main-island are genetically homogeneous [14]; therefore, we think there is no evidence for population heterogeneity between the discovery stage and the replication stage.

We further performed a GWAS meta-analysis for diabetic nephropathy including patients with microalbuminuria as cases, and found that rs56094641 in *FTO* was significantly associated with diabetic nephropathy also in this analysis (*P* = 2.40 × 10^−9^, OR = 1.18, 95% CI 1.15−1.31, S5 and S6 Tables). However, the association was not stronger than that in the analysis using overt nephropathy as cases, suggesting the *FTO* variant was associated with advanced stages of diabetic nephropathy.

Our study has some limitations. Firstly, the detail clinical information, i.e. blood pressure, Hemoglobin A1c, lipid profiles, prescription of antihypertensive treatments, was not available in many participants in Stage-1 samples. It means that we cannot exclude the possibility that the association of the SNP with diabetic nephropathy is mediated by some risk factors except age, sex and BMI. Second, by *in silico* replication using SUMMIT consortium data for type 2 diabetes, the association of *FTO* variants with diabetic nephropathy was not replicated in European patients with type 2 diabetes (Table 3, S7 Table), and the association of *FTO* variants with diabetic nephropathy showed a genome-wide significant association only in a joint analysis for the discovery stage; therefore, these observations may not be in line with standards for GWA studies; further study, such as a large-scaled longitudinal study, is required to elucidate the association of *FTO* locus with diabetic nephropathy.

## Conclusions

We performed GWAS for diabetic nephropathy in Japanese patients with type 2 diabetes. One SNP locus, *FTO* locus, showed a significant association with susceptibility to diabetic nephropathy, and the association attained a genome-wide significant level. Further studies are required to confirm the association of this locus with diabetic nephropathy.

## Supporting information

S1 Fig Results of principal component analysis in stage-1 and stage-2. (PDF)

S2 Fig Quantile-quantile plot. A: Stage-1, set-1, B: Stage-1, set-2.
(PDF)

S3 Fig Regional plot of each candidate locus. Results of stage-1 GWAS meta-analysis are shown. Red, diamond-shaped plots indicate the most significant variants in each locus after combining stage 1 and stage 2 data. *r*^2^, linkage disequilibrium coefficient; chr., chromosome.
(PDF)

S1 Table SNPs with p values < 10^−4^ in the discovery stage. (PDF)

S2 Table Candidate SNP loci for overt diabetic nephropathy in the discovery stage. (PDF)

S3 Table Effects of the adjustment for age, sex and BMI on association of the *FTO* locus with susceptibility to diabetic nephropathy. (PDF)

S4 Table Association of previously reported loci with overt diabetic nephropathy in the stage 1 analysis. (PDF)

S5 Table Six SNP loci associated with diabetic nephropathy including microalbuminuria (Meta-analysis in Discovery Stage, P < 5 x 10^−7^) in Japanese patients with type 2 diabetes. (PDF)

S6 Table Six SNP loci associated with diabetic nephropathy including microalbuminuria (Meta-analysis in Discovery Stage, P<5x10^-7^) in Japanese patients with type 2 diabetes. (PDF)

S7 Table In silico replication of candidate loci for Diabetic Nephropathy in the data from the SUMMIT Consortium. (PDF)

S8 Table Contributors for SUMMIT consortium. (PDF)

S1 File Meta-analysis-on-genetic-association-studies-form. (DOCX)
