# Supplementary figures and images for: A variant within the FTO confers susceptibility to diabetic nephropathy in Japanese patients with type 2 diabetes
Source: PLoS One. 2018 Dec 19;13(12):e0208654. doi: 10.1371/journal.pone.0208654 (PMC6300288; doi:10.1371/journal.pone.0208654)

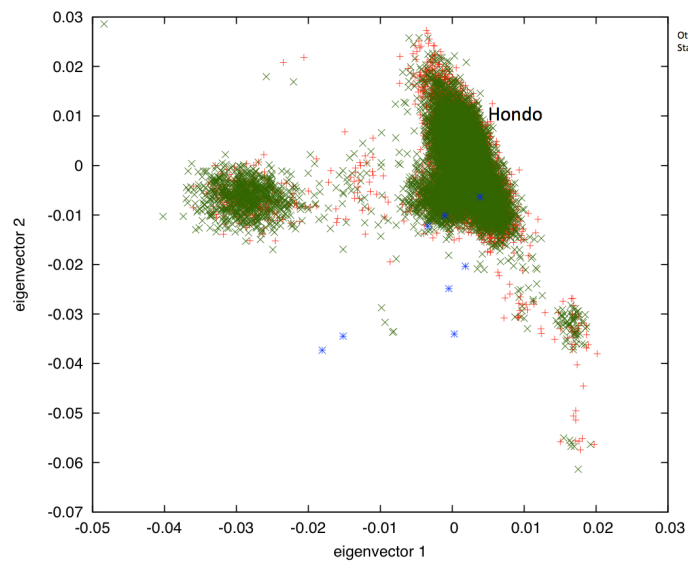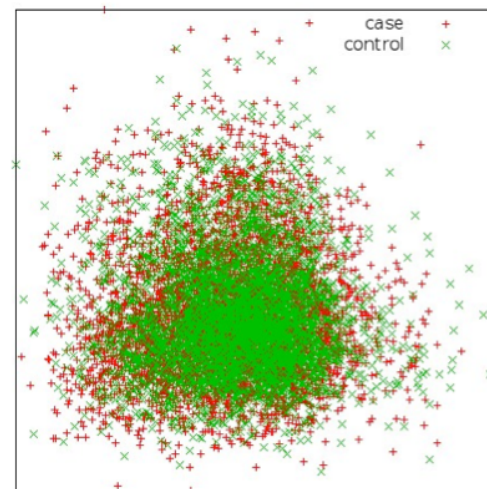

Stage-1

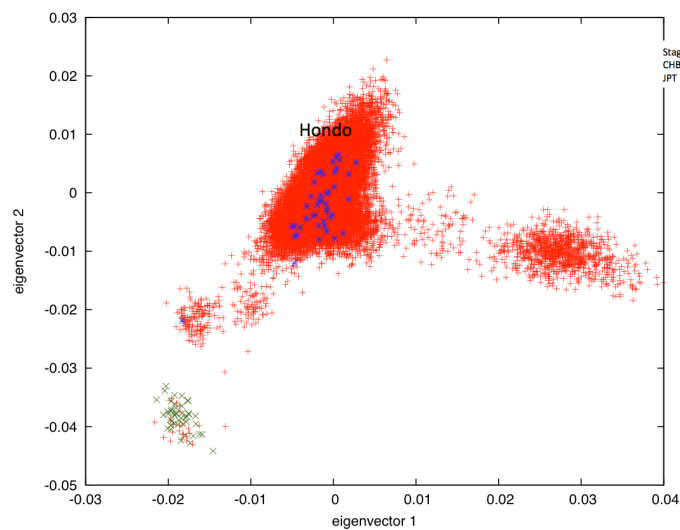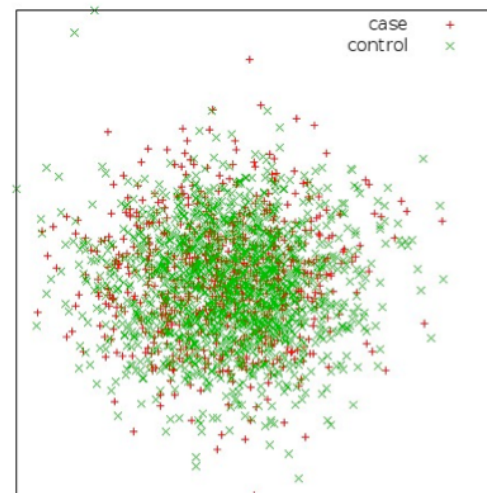

Stage-2

S1 Fig. Results of principal component analysis in stage-1 and stage-2

Supplement: S1 Fig — (PDF) [file pone.0208654.s001.pdf]
