# Supplementary material for: A variant within the FTO confers susceptibility to diabetic nephropathy in Japanese patients with type 2 diabetes
Source: PLoS One. 2018 Dec 19;13(12):e0208654. doi: 10.1371/journal.pone.0208654 (PMC6300288; doi:10.1371/journal.pone.0208654)
Supplement: S2 Fig — A: Stage-1, set-1, B: Stage-1, set-2. (PDF) [file pone.0208654.s002.pdf]

A: Stage-1,set-1 (RSQ>0.7)
6,000,374 SNPs
Lambda GC =1.043420

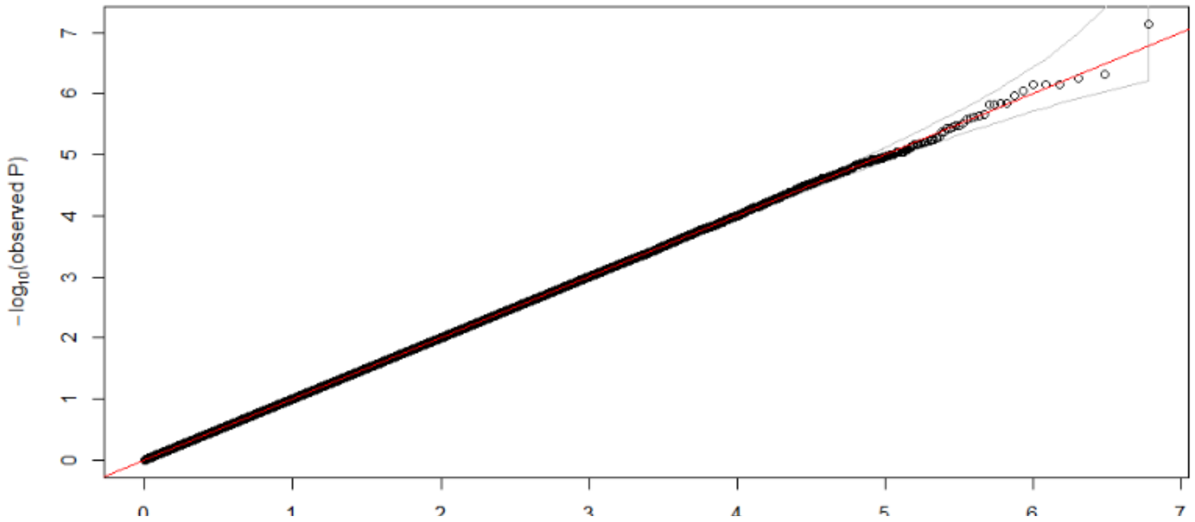

B: Stage-1,set-2 (RSQ>0.7)
5,894,225 SNPs
Lambda GC =1.001204

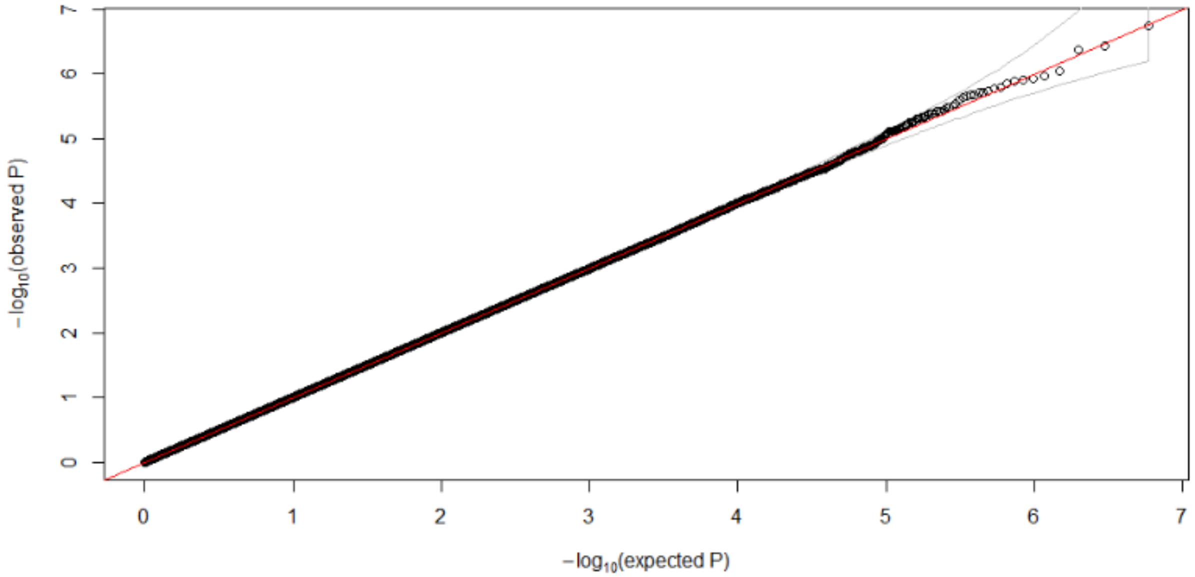

S2 Fig. Quantile-quantile plot
A: Stage-1, set-1 B: Stage-1, set-2
