# Supplementary material for: A variant within the FTO confers susceptibility to diabetic nephropathy in Japanese patients with type 2 diabetes
Source: PLoS One. 2018 Dec 19;13(12):e0208654. doi: 10.1371/journal.pone.0208654 (PMC6300288; doi:10.1371/journal.pone.0208654)
Supplement: S3 Fig — Results of stage-1 GWAS meta-analysis are shown. Red, diamond-shaped plots indicate the most significant variants in each locus after combining stage 1 and stage 2 data. r2, linkage disequilibrium coefficient; chr., chromosome. (PDF) [file pone.0208654.s003.pdf]

S2 Fig.: Regional plot of each candidate locus.
Results of stage-1 GWAS meta-analysis are shown. Red, diamond-shaped plots indicate the most significant variants in each locus after combining stage 1 and stage 2 data. r^2, linkage disequilibrium coefficient; chr., chromosome.

a) rs56094641

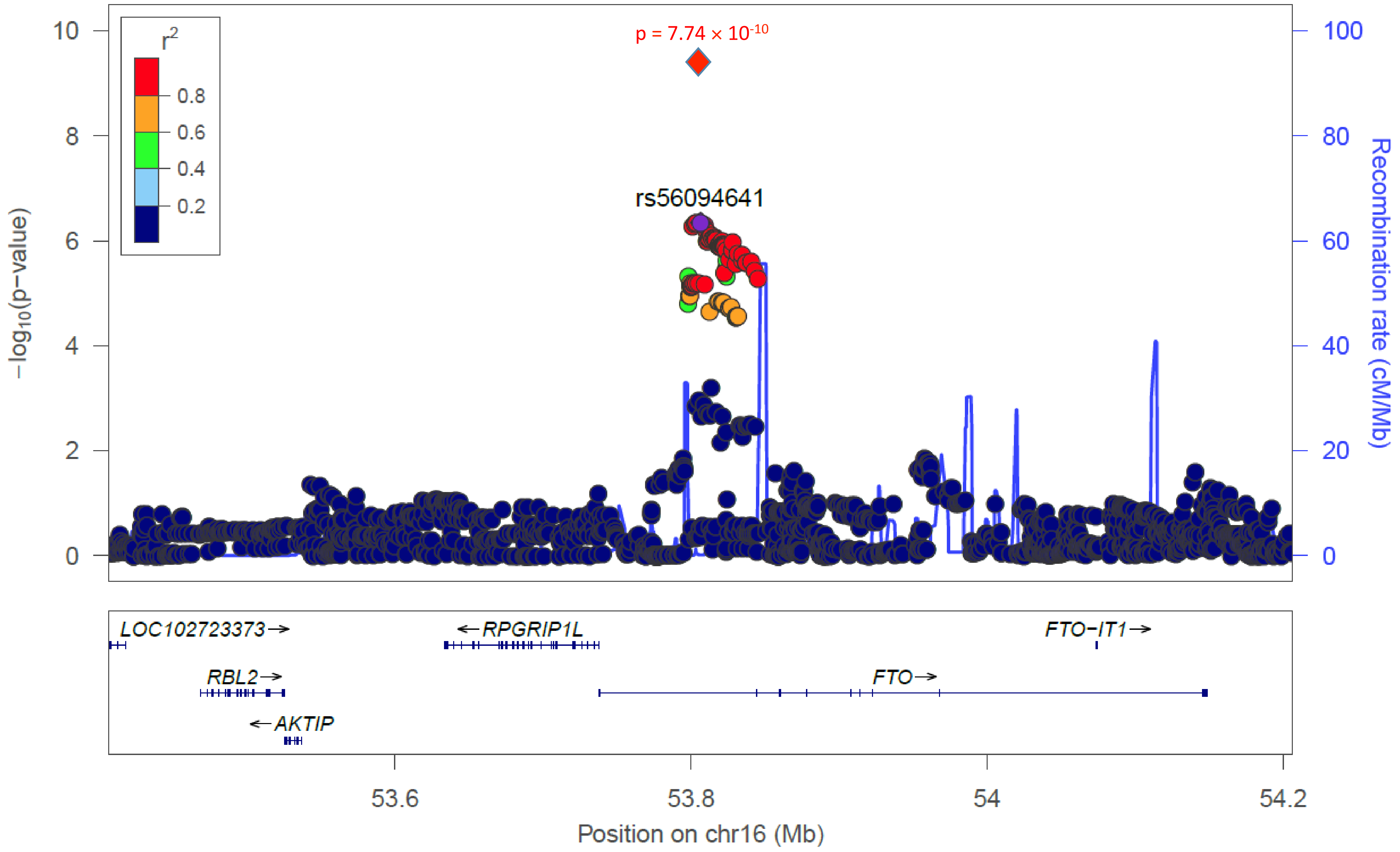

b) rs895157

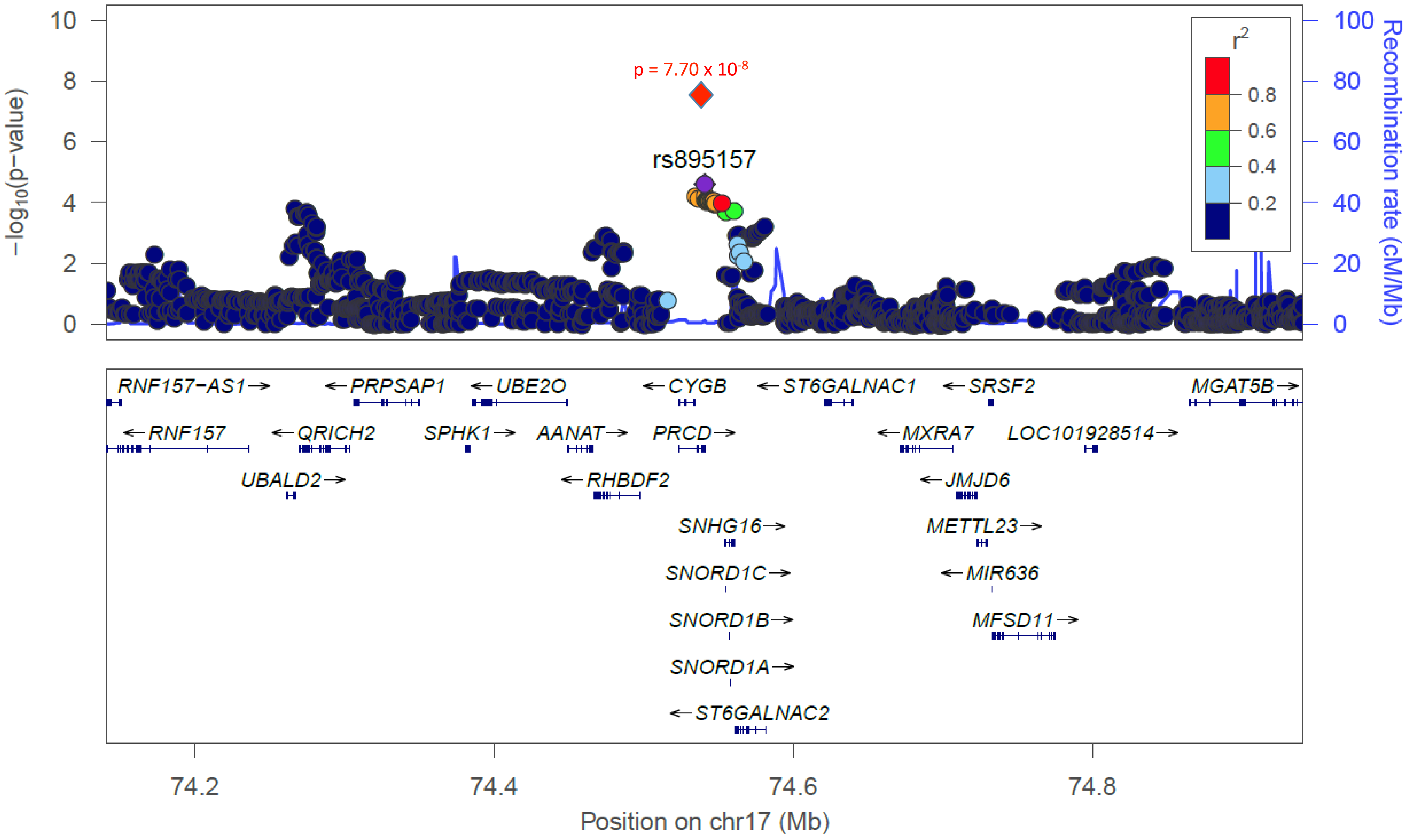

p = 7.70 x 10-8
rs895157
r2
0.8
0.6
0.4
0.2
−log10(p−value)
Recombination rate (cM/Mb)
10
8
6
4
2
0
100
80
60
40
20
0
RNF157−AS1→
←PRPSAP1
←UBE2O
←CYGB
←ST6GALNAC1
←SRSF2
MGAT5B→
←RNF157
←QRICH2
SPHK1→
AANAT→
PRCD→
←MXRA7
LOC101928514→
UBALD2→
←RHBDF2
←JMJD6
SNHG16→
METTL23→
SNORD1C→
←MIR636
SNORD1B→
MFSD11→
SNORD1A→
←ST6GALNAC2
74.2
74.4
74.6
74.8
Position on chr17 (Mb)

c) rs10144968

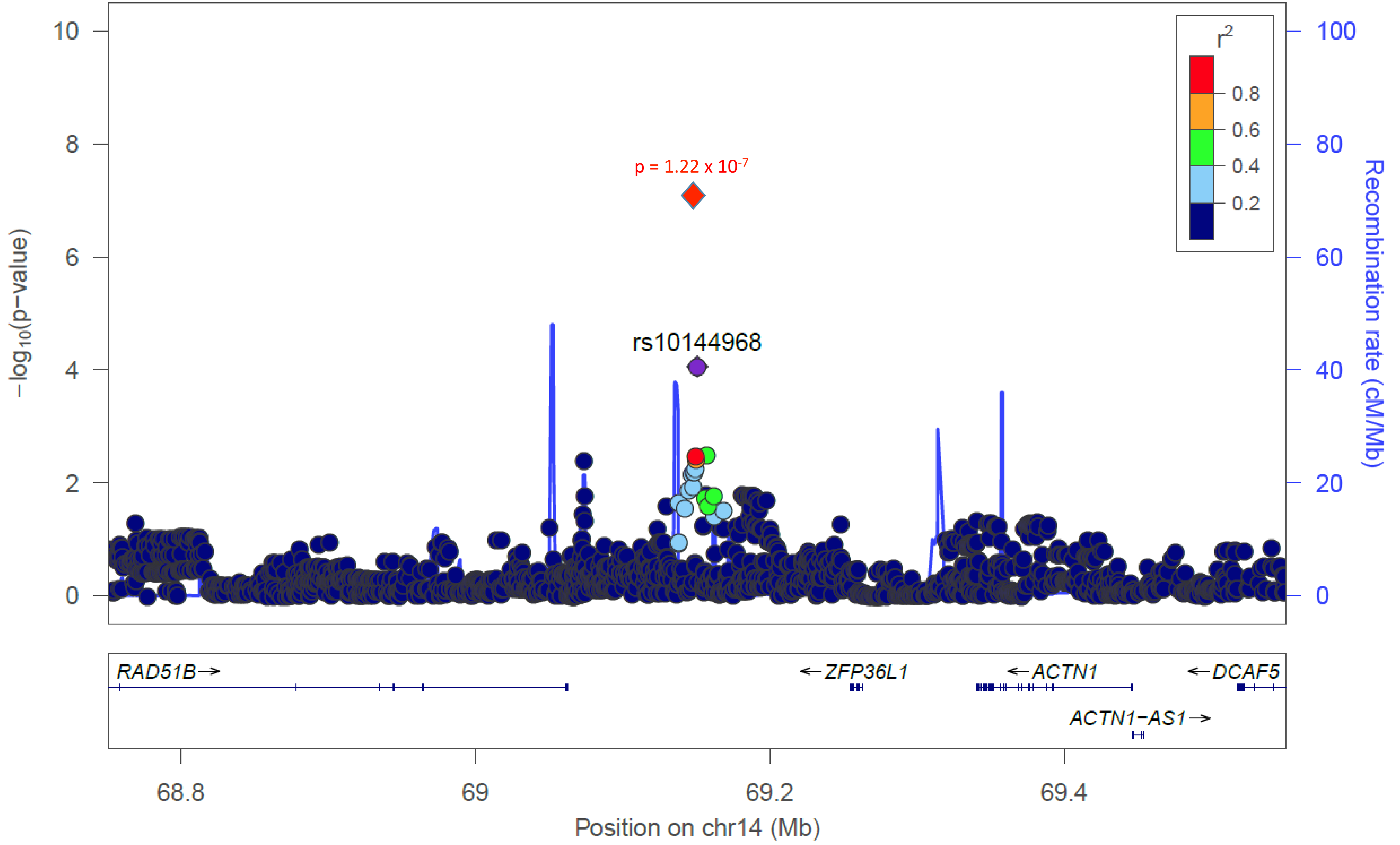

d) rs13306536

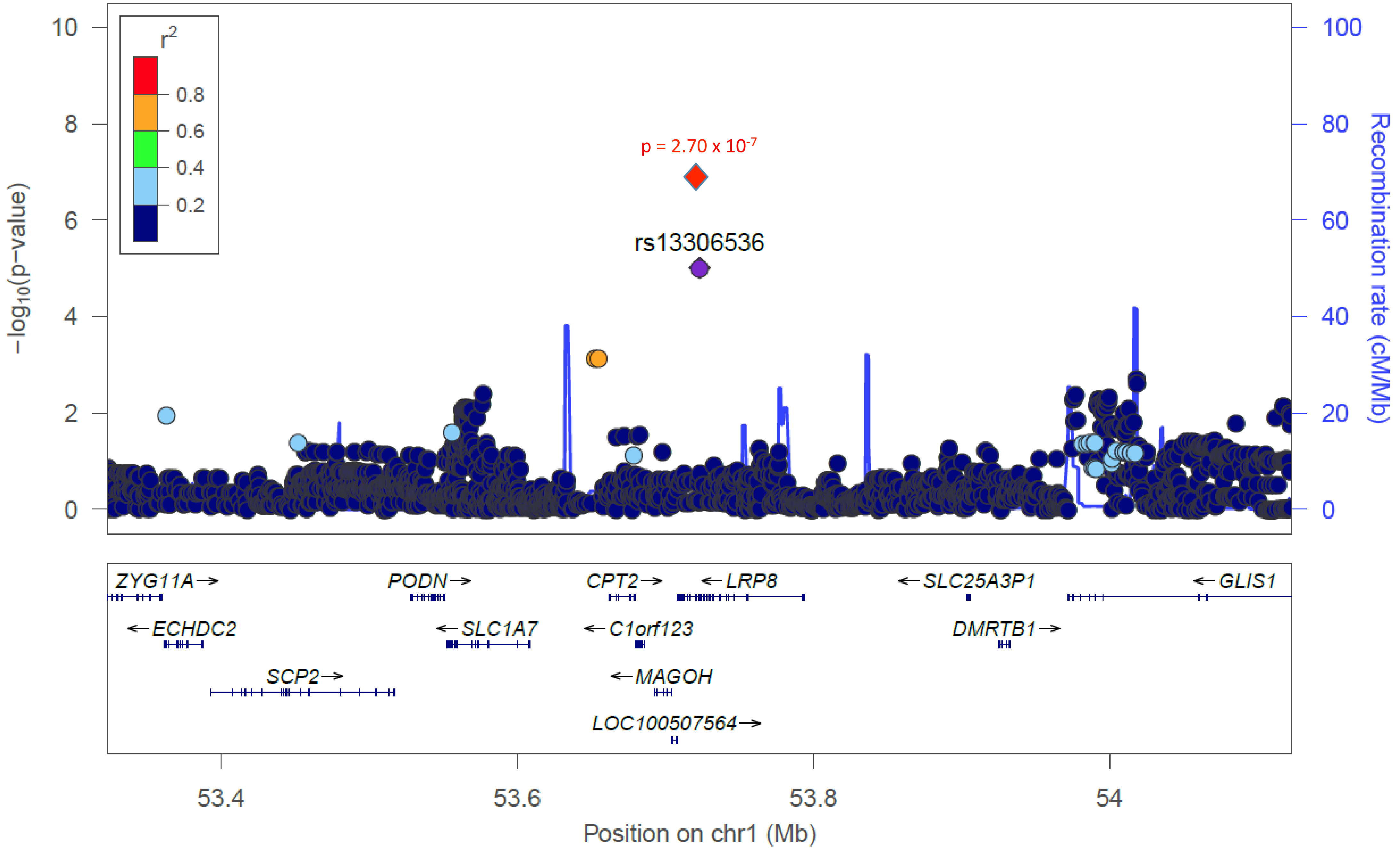

e) rs7544082

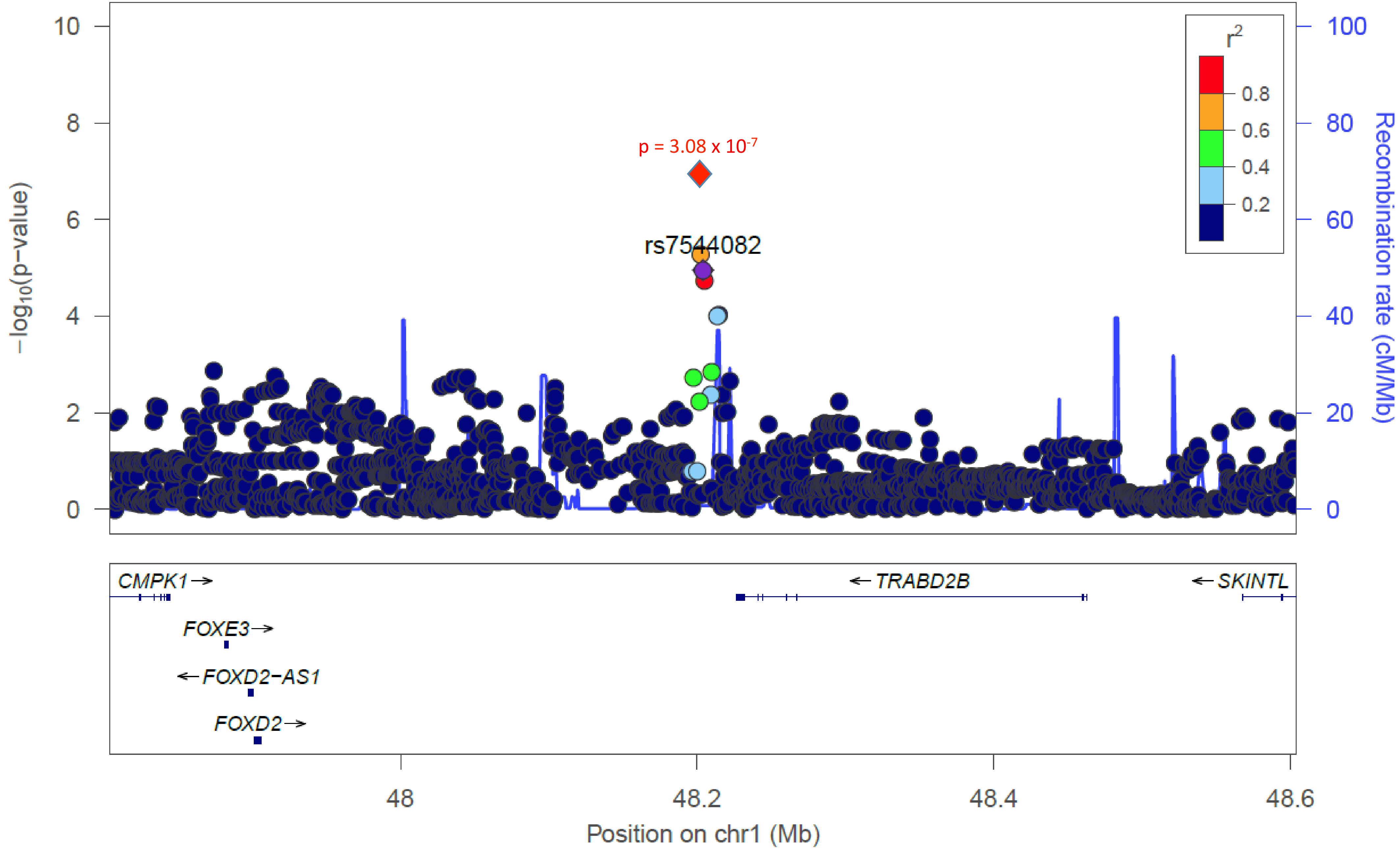

p = 3.08 x 10-7
rs7544082
r2
0.8
0.6
0.4
0.2
10
8
6
4
2
0
−log10(p−value)
100
80
60
40
20
0
Recombination rate (cM/Mb)
CMPK1→
FOXE3→
←FOXD2−AS1
FOXD2→
←TRABD2B
←SKINTL
48
48.2
48.4
48.6
Position on chr1 (Mb)

f) rs11101179

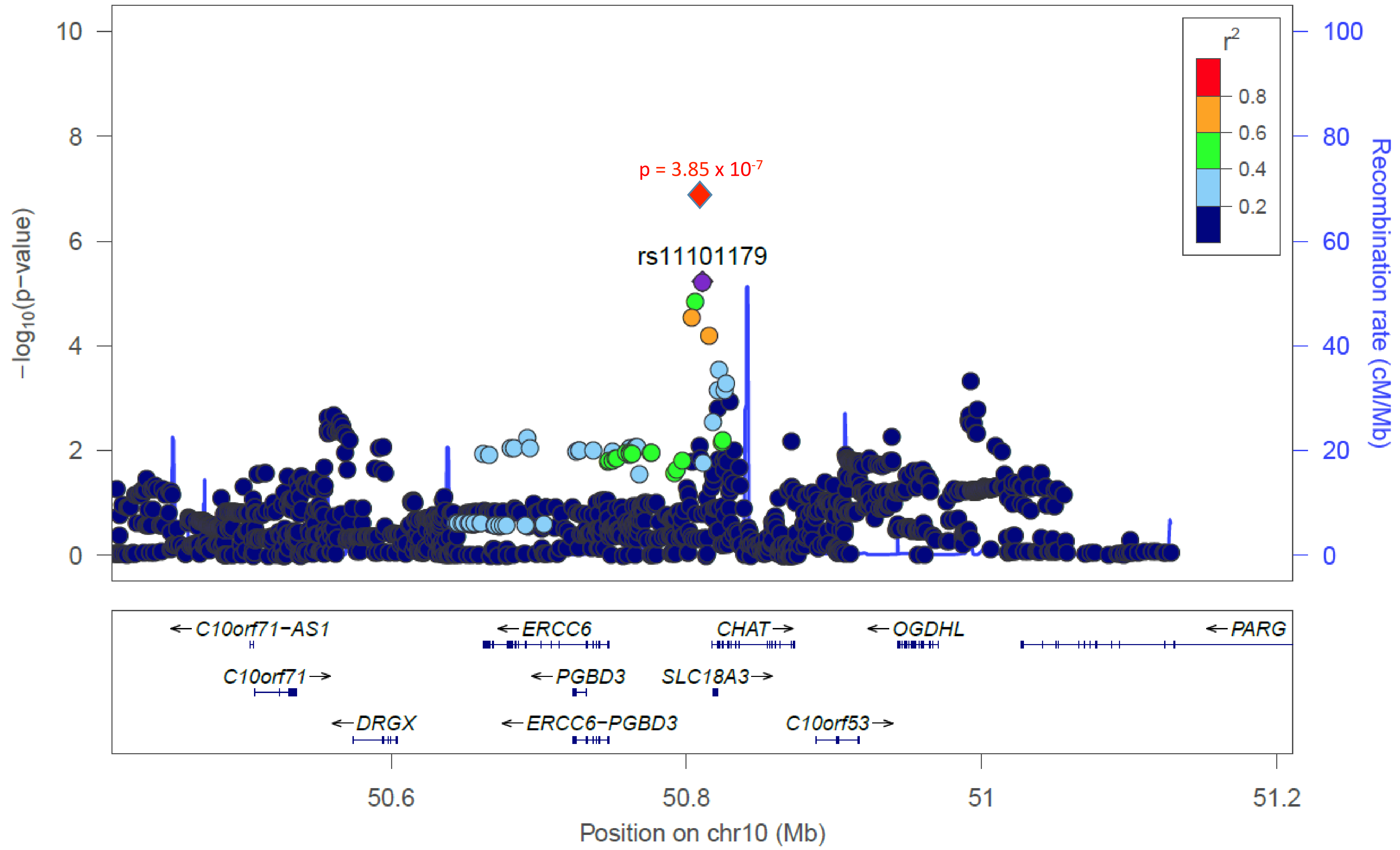

g) rs710375

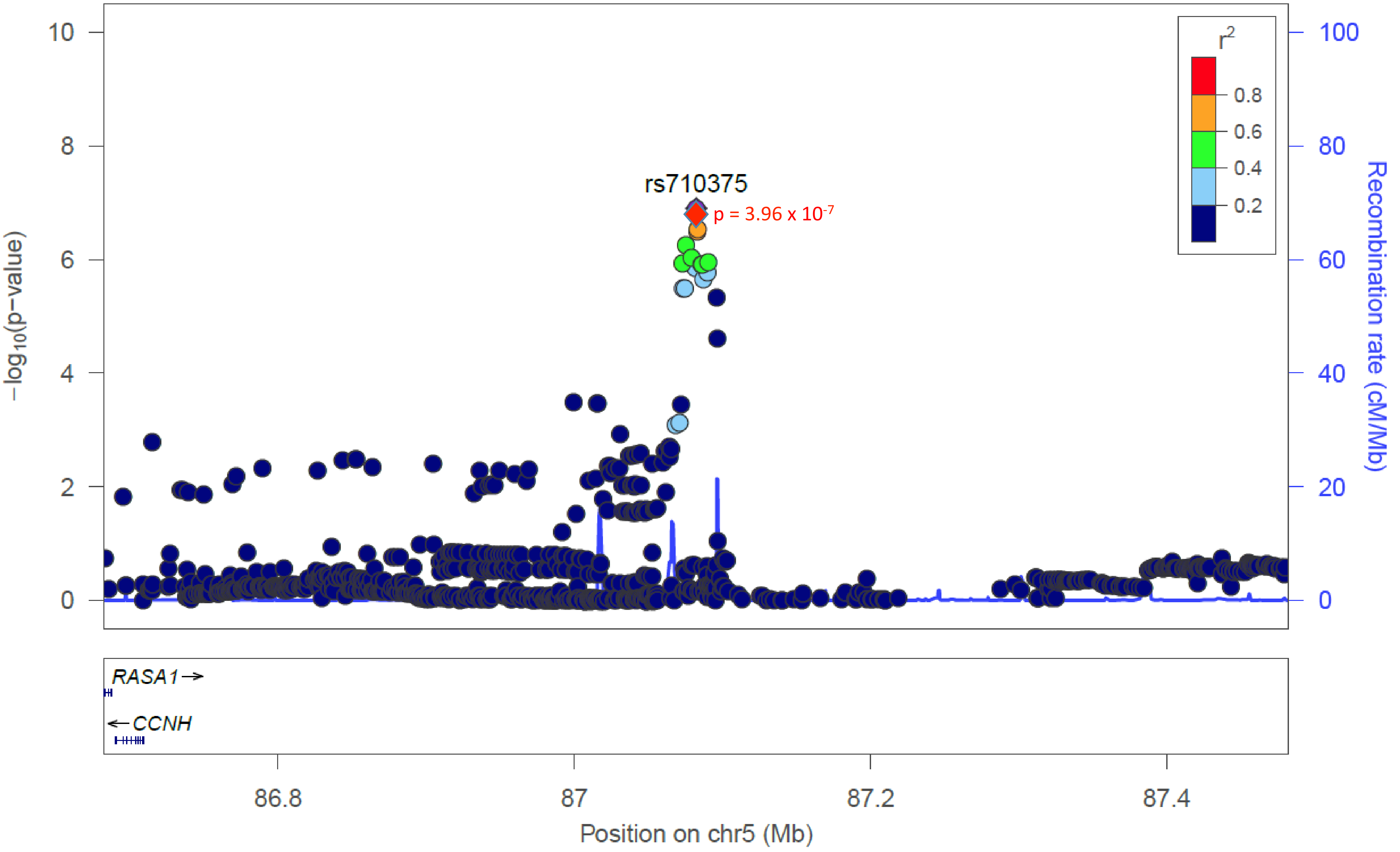

rs710375
p = 3.96 x 10-7
r2
0.8
0.6
0.4
0.2
−log10(p−value)
Recombination rate (cM/Mb)
10
8
6
4
2
0
100
80
60
40
20
0
RASA1→
←CCNH
86.8
87
87.2
87.4
Position on chr5 (Mb)
