# Supplementary material for: A variant within the FTO confers susceptibility to diabetic nephropathy in Japanese patients with type 2 diabetes
Source: PLoS One. 2018 Dec 19;13(12):e0208654. doi: 10.1371/journal.pone.0208654 (PMC6300288; doi:10.1371/journal.pone.0208654)
Supplement: S1 Table — (PDF) [file pone.0208654.s004.pdf]

S1 Table: SNPs with p values < 10^{-4} in the discovery stage

SNPID	OR	95% CI Lower	95% CI Upper	p-value	Effect allele	non-effect Allele	r^2 in Stage-1 set-1	r^2 in Stage-1 set-2	chr	position (GRCh37.p13)	*Nearest Gene*
rs1887586	1.14212	1.0686	1.22069	9.06E-05	T	C	0.9517	0.9463	1	42825059	*RIMKLA*
rs4927013	0.84329	0.783784	0.907306	4.98E-06	T	A	0.7688	0.9052	1	48202266	*LOC388630*
rs7544082	0.85256	0.794036	0.915406	1.10E-05	C	A	0.8118	0.9986	1	48203990	*LOC388630*
rs34640822	0.85579	0.79704	0.918869	1.77E-05	T	C	0.8265	0.9898	1	48204857	*LOC388630*
rs2165193	0.87813	0.822521	0.937504	9.89E-05	T	C	0.9894	0.767	1	48213641	*LOC388630*
rs17103715	0.87044	0.811974	0.93312	9.18E-05	C	T	0.8413	0.9926	1	48214623	*LOC388630*
rs13306536	0.75723	0.669467	0.856505	9.67E-06	C	T	0.9144	0.8541	1	53723222	*LRP8*
rs1323826	0.87227	0.815602	0.932877	6.68E-05	A	G	0.9965	0.9246	1	58232509	*DAB1*
rs6703218	1.2146	1.10897	1.33029	2.81E-05	C	T	0.9971	0.9987	1	64868506	*CACHD1*
rs6661844	1.21813	1.11212	1.33425	2.16E-05	G	T	0.9949	0.9952	1	64870696	*CACHD1*
rs1514968	1.21596	1.11014	1.33187	2.56E-05	G	A	0.9942	0.9915	1	64874759	*CACHD1*
rs55802864	1.21621	1.11036	1.33214	2.51E-05	A	C	0.9938	0.9911	1	64878757	*CACHD1*
rs61784524	1.21801	1.10981	1.33676	3.25E-05	C	T	0.9725	0.9752	1	64880167	*CACHD1*
rs61784525	1.22878	1.11754	1.35109	2.09E-05	G	C	0.9152	0.9204	1	64880221	*CACHD1*
rs9725347	1.30225	1.14484	1.4813	5.87E-05	G	A	0.727	0.7383	1	64880656	*CACHD1*
rs192176527	1.2241	1.10895	1.3512	6.03E-05	C	T	0.9159	0.9119	1	64881505	*CACHD1*
rs57330035	1.21167	1.1041	1.32972	5.17E-05	G	A	0.9867	0.989	1	64882039	*CACHD1*
rs11808174	1.19442	1.09848	1.29874	3.20E-05	G	A	0.9647	0.9801	1	64884113	*CACHD1*
rs11590953	1.21191	1.10432	1.32999	5.08E-05	A	G	0.9869	0.9892	1	64885759	*CACHD1*
rs7533312	1.1821	1.08743	1.28502	8.57E-05	C	G	0.9905	0.9919	1	64891785	*CACHD1*
rs11208448	1.20369	1.09881	1.31859	6.73E-05	A	G	0.9982	0.9986	1	64892397	*CACHD1*
rs11208449	1.18117	1.08657	1.28401	9.26E-05	A	G	0.9904	0.992	1	64893492	*CACHD1*
rs11208450	1.18047	1.08585	1.28333	9.93E-05	C	T	0.9739	0.9599	1	64895633	*CACHD1*
rs12025177	1.20647	1.09936	1.32402	7.59E-05	A	T	0.9356	0.9532	1	64900424	*CACHD1*
rs71664889	0.82529	0.754075	0.903237	3.04E-05	T	C	0.8988	0.6842	1	115939165	*NGF*
rs17163886	0.8282	0.758143	0.904733	2.91E-05	A	C	0.9162	0.7044	1	115956697	*NGF*
rs17163888	0.82973	0.75954	0.9064	3.48E-05	A	G	0.9235	0.7054	1	115957458	*NGF*
rs11488625	0.82973	0.75954	0.9064	3.48E-05	T	A	0.9245	0.7061	1	115959226	*NGF*
rs34213778	0.82986	0.759661	0.906544	3.54E-05	A	C	0.9268	0.7063	1	115962048	*NGF*
rs35549615	0.83137	0.761079	0.908156	4.18E-05	G	A	0.9329	0.7082	1	115964566	*NGF*
rs35757399	0.83137	0.761079	0.908156	4.18E-05	G	A	0.9332	0.7084	1	115964749	*NGF*
rs11808454	1.356	1.19066	1.5443	4.43E-06	C	G	0.8259	0.8249	1	201396107	*TNNI1*
rs13374832	1.25914	1.12884	1.4045	3.56E-05	G	A	0.9093	0.8931	1	201400917	*TNNI1*
rs75427337	1.52593	1.26255	1.84425	1.23E-05	G	A	0.7258	0.7049	1	201401316	*TNNI1*
rs6672661	1.14807	1.07233	1.22916	7.32E-05	G	A	0.9497	0.9993	1	203201256	*CHIT1*
rs1340240	1.16835	1.08916	1.2533	1.39E-05	C	T	0.972	0.9928	1	203202644	*CHIT1*
rs6658255	1.1668	1.08772	1.25163	1.65E-05	A	G	0.969	0.9849	1	203204169	*CHIT1*
rs16851157	1.17616	1.09644	1.26167	5.87E-06	C	G	0.9841	0.9871	1	203207021	*CHIT1*
rs16851159	1.17641	1.09856	1.25978	3.31E-06	A	G	0.9925	0.9955	1	203207621	*CHIT1*
rs10920589	1.14305	1.07131	1.21959	5.28E-05	G	T	0.9994	0.9969	1	203208696	*CHIT1*
rs59085380	1.17641	1.09856	1.25978	3.31E-06	T	C	0.9948	0.9963	1	203208785	*CHIT1*
rs7547716	1.1716	1.09219	1.25678	9.75E-06	T	G	1	1	1	203209481	*CHIT1*
rs55950560	1.17264	1.09316	1.2579	8.69E-06	C	T	0.9997	0.9996	1	203210385	*CHIT1*
rs55981609	1.17264	1.09316	1.2579	8.69E-06	A	G	0.9994	0.9994	1	203210422	*CHIT1*
rs76719424	1.1502	1.07408	1.23171	6.18E-05	G	T	0.9598	0.9612	1	203243203	*LOC100506775*
rs2185400	1.15118	1.07507	1.23267	5.48E-05	A	G	0.9997	0.9995	1	203243600	*LOC100506775*
rs7551146	1.15118	1.07507	1.23267	5.48E-05	C	T	0.9999	0.9998	1	203244113	*LOC100506775*
rs74137603	1.15092	1.07484	1.2324	5.63E-05	G	T	0.9997	0.9986	1	203245120	*LOC100506775*
rs1106832	1.15297	1.07675	1.23459	4.52E-05	T	C	0.9946	0.9967	1	203245821	*LOC100506775*
rs2486932	1.15426	1.07787	1.23605	4.01E-05	C	T	0.9959	0.9883	1	203247003	*LOC100506775*
rs2066023	1.18602	1.09285	1.28713	4.37E-05	G	A	0.9884	0.9065	1	211301058	*KCNH1*
rs3811395	1.21501	1.11735	1.3212	5.23E-06	G	A	0.9991	0.8981	1	211308116	*KCNH1*
rs11119680	1.18952	1.09615	1.29085	3.17E-05	G	C	0.9968	0.9088	1	211309203	*KCNH1*
rs11119687	1.17195	1.08233	1.269	9.27E-05	C	T	0.9943	0.9831	1	211325227	*KCNH1*
rs7551205	1.17141	1.08191	1.26832	9.57E-05	G	A	0.9948	0.9923	1	211326781	*KCNH1*
rs7547206	1.17034	1.0827	1.26508	7.47E-05	A	G	0.9987	0.9976	1	211328573	*RPS25P2*
rs11119690	1.17059	1.08293	1.26535	7.30E-05	C	A	0.9995	0.9986	1	211334211	*RPS25P2*

rs1338351	1.17072	1.08305	1.26548	7.22E-05	C	T	0.9994	0.9997	1	211339761	*RPS25P2*
rs11119697	1.17072	1.08305	1.26548	7.22E-05	T	C	0.9995	1	1	211340622	*RPS25P2*
rs7541861	1.17072	1.08305	1.26548	7.22E-05	T	G	0.9995	1	1	211341721	*RPS25P2*
rs11119698	1.17072	1.08305	1.26548	7.22E-05	C	A	0.9996	1	1	211342286	*RPS25P2*
rs12036302	1.17072	1.08305	1.26548	7.22E-05	A	C	1	1	1	211344081	*RPS25P2*
rs1933523	1.17059	1.08293	1.26535	7.30E-05	G	A	0.9999	0.9995	1	211344683	*RPS25P2*
rs6663683	1.17151	1.08379	1.26634	6.72E-05	T	A	0.998	0.9968	1	211348493	*RPS25P2*
rs7525019	1.17034	1.0827	1.26508	7.47E-05	T	A	0.9998	0.9974	1	211350242	*RPS25P2*
rs77746757	1.24173	1.11362	1.38457	9.73E-05	C	G	0.9926	0.9891	2	1788730	*MYT1L*
rs10174926	1.24145	1.11337	1.38426	9.90E-05	T	C	0.9923	0.9811	2	1789294	*MYT1L*
rs10185580	1.24145	1.11337	1.38426	9.90E-05	C	T	0.992	0.9807	2	1789330	*MYT1L*
rs13000148	1.23236	1.11914	1.35702	2.14E-05	C	T	0.8779	0.9194	2	26526763	*GPR113*
rs4665850	1.22744	1.11668	1.34919	2.17E-05	C	T	0.9284	0.9455	2	26527220	*GPR113*
rs1965093	1.188	1.09082	1.29384	7.61E-05	T	C	0.9331	0.9662	2	26530018	*GPR113*
rs2007556	1.21777	1.11201	1.33358	2.14E-05	T	G	0.9995	0.9998	2	26532491	*GPR113*
rs3856397	1.18315	1.08832	1.28624	7.97E-05	A	G	0.9879	0.9981	2	26532735	*GPR113*
rs3851967	1.18242	1.08765	1.28545	8.46E-05	T	C	0.9931	0.9869	2	26533023	*GPR113*
rs7568529	1.21356	1.10603	1.33154	4.33E-05	A	G	0.9767	0.9762	2	26534801	*GPR113*
rs10865401	1.18208	1.08727	1.28516	8.81E-05	T	G	0.9981	0.9792	2	26535342	*GPR113*
rs11126434	1.18208	1.08727	1.28516	8.81E-05	G	T	0.9984	0.9788	2	26535363	*GPR113*
rs10865402	1.18208	1.08727	1.28516	8.81E-05	G	A	0.9981	0.9785	2	26535383	*GPR113*
rs118080383	0.79432	0.712485	0.88555	3.31E-05	G	A	0.9555	0.961	2	62784619	*RSL24D1P2*
rs6547314	0.82026	0.746975	0.900734	3.33E-05	C	A	0.9976	0.8909	2	80749784	*CTNNA2*
rs6729523	0.82005	0.746748	0.900551	3.29E-05	A	G	0.9979	0.8921	2	80750869	*CTNNA2*
rs11886929	0.6908	0.57634	0.827987	6.27E-05	G	A	0.7554	0.7544	2	123778282	*LOC100422580*
rs17009201	0.691	0.576552	0.828173	6.32E-05	G	A	0.7563	0.7554	2	123778618	*LOC100422580*
rs78000774	0.69116	0.576681	0.828357	6.38E-05	G	T	0.7593	0.7586	2	123787518	*LOC100422580*
rs150448302	0.69123	0.576745	0.828449	6.41E-05	G	A	0.7596	0.7589	2	123789387	*LOC100422580*
rs114599051	0.69146	0.576937	0.828726	6.51E-05	A	C	0.7603	0.7605	2	123792461	*LOC100422580*
rs57116385	0.69154	0.577001	0.828818	6.55E-05	C	T	0.7612	0.7616	2	123796249	*LOC100422580*
rs1395927	0.69223	0.577579	0.829647	6.85E-05	C	T	0.7615	0.7618	2	123797629	*LOC100422580*
rs185029049	0.70925	0.600314	0.837964	5.40E-05	C	T	0.8129	0.8101	2	123830335	*LOC100422580*
rs534830	0.69221	0.575207	0.833018	9.87E-05	T	C	0.9788	0.9717	2	137176301	*UBBP1*
rs6746511	1.17881	1.08601	1.27953	8.41E-05	C	T	0.9436	0.892	2	224039566	*KCNE4*
rs9855025	0.8237	0.752828	0.90124	2.39E-05	T	C	0.9967	0.8894	3	5434207	*MRPS35P1*
rs7625401	1.28648	1.1352	1.45791	7.92E-05	G	C	0.8093	0.881	3	10210942	*IRAK2*
rs142390470	1.25562	1.12173	1.40549	7.60E-05	G	A	0.9939	0.9784	3	10218223	*IRAK2*
rs61089659	1.25795	1.12366	1.40829	6.78E-05	C	T	0.9852	0.9604	3	10223033	*IRAK2*
rs75851138	1.26496	1.12782	1.41878	5.96E-05	C	T	0.9559	0.9275	3	10223370	*IRAK2*
rs6550928	1.31479	1.14571	1.50883	9.75E-05	T	G	0.9462	0.9281	3	25007199	*CFL1P7*
rs2362766	1.34889	1.16182	1.56609	8.53E-05	A	C	0.9282	0.9746	3	25007658	*CFL1P7*
rs4858673	1.35032	1.16323	1.5675	7.91E-05	G	C	0.9301	0.9967	3	25008633	*CFL1P7*
rs111235326	1.16676	1.08348	1.25643	4.46E-05	A	G	0.7595	0.8096	3	69038756	*C3orf64*
rs36138181	1.16368	1.08257	1.25088	3.92E-05	T	C	0.7947	0.8403	3	69038777	*C3orf64*
rs66614734	1.16989	1.08639	1.2598	3.28E-05	G	A	0.7747	0.8061	3	69038792	*C3orf64*
rs77015412	0.7245	0.619232	0.847652	5.74E-05	G	C	0.7899	0.7503	3	99414163	*COL8A1*
rs74445365	0.68679	0.578836	0.814866	1.66E-05	T	C	0.7497	0.7033	3	99439469	*COL8A1*
rs183695838	0.51376	0.369216	0.714891	7.78E-05	A	G	0.7573	0.7533	3	150958368	*MED12L*
rs190929035	0.49843	0.359482	0.691072	2.96E-05	T	C	0.7426	0.7453	3	150968079	*MED12L*
rs182840241	0.76512	0.679545	0.861476	9.70E-06	T	G	0.7095	0.7388	3	151086520	*MED12L*
rs150285637	0.7656	0.679659	0.862396	1.10E-05	T	G	0.8012	0.8052	3	151086530	*MED12L*
rs17802266	1.27403	1.13281	1.43285	5.33E-05	C	A	0.9971	0.9969	4	1158991	*SPON2*
rs78500739	1.31969	1.15372	1.50953	5.23E-05	G	A	0.8446	0.8101	4	1169631	*SPON2*
rs117650098	0.59822	0.464856	0.769857	6.54E-05	G	T	0.89	0.8529	4	95549420	*PDLIM5*
rs7671545	1.16136	1.0866	1.24126	1.05E-05	C	T	0.9985	0.9991	4	95947520	*BMPR1B*
rs10516957	1.15745	1.08287	1.23717	1.69E-05	T	C	0.9998	0.9998	4	95949206	*BMPR1B*
rs13134763	1.16071	1.086	1.24057	1.13E-05	A	C	0.9953	0.995	4	95953644	*BMPR1B*
rs13134993	1.16071	1.086	1.24057	1.13E-05	A	G	0.9954	0.995	4	95953741	*BMPR1B*
rs1816461	1.15981	1.08515	1.2396	1.26E-05	A	G	0.999	0.9997	4	95954980	*BMPR1B*
rs13134042	1.16066	1.08587	1.2406	1.17E-05	G	A	0.9963	0.9902	4	95959027	*BMPR1B*
rs9997720	0.86545	0.809802	0.924928	2.03E-05	A	G	0.9949	0.9898	4	95963792	*BMPR1B*
rs184544569	1.16253	1.0856	1.24491	1.63E-05	C	T	0.9695	0.9614	4	95964319	*BMPR1B*

rs4699408	1.16214	1.08725	1.24218	9.80E-06	C	G	0.9883	0.9886	4	95982863	*BMPR1B*
rs1836260	1.16368	1.0887	1.24383	8.17E-06	G	A	0.9947	0.9954	4	95988614	*BMPR1B*
rs13152580	1.16555	1.09037	1.24592	6.69E-06	C	G	0.979	0.9804	4	96004182	*BMPR1B*
rs1897810	1.16818	1.09283	1.24872	4.89E-06	A	G	0.9935	0.9965	4	96016055	*BMPR1B*
rs13103264	1.16713	1.09185	1.24761	5.55E-06	G	A	0.9882	0.9951	4	96019477	*BMPR1B*
rs146755264	3.44737	1.86196	6.38272	8.22E-05	C	A	0.8429	0.7652	4	96477719	*UNC5C*
rs75566894	3.44337	1.85988	6.37505	8.34E-05	G	C	0.8437	0.7662	4	96477918	*UNC5C*
rs76671526	3.45648	1.88009	6.35463	6.55E-05	A	G	0.8617	0.774	4	96483630	*UNC5C*
rs113077462	3.31509	1.8232	6.02775	8.54E-05	A	G	0.8507	0.7721	4	96486517	*UNC5C*
rs72878325	3.31017	1.82405	6.00708	8.26E-05	C	T	0.8514	0.7725	4	96487005	*UNC5C*
rs76968807	3.302	1.82311	5.98057	8.10E-05	C	T	0.8523	0.7734	4	96487813	*UNC5C*
rs78171761	3.29605	1.82031	5.96818	8.24E-05	T	A	0.8533	0.7748	4	96488195	*UNC5C*
rs114361053	3.28494	1.81797	5.93565	8.14E-05	C	T	0.8549	0.7778	4	96488504	*UNC5C*
rs74446244	3.27263	1.81504	5.90078	8.08E-05	C	T	0.8568	0.7819	4	96490160	*UNC5C*
rs76102424	3.26376	1.81374	5.87302	7.94E-05	C	G	0.8582	0.7852	4	96491349	*UNC5C*
rs79422810	3.25645	1.81002	5.85876	8.15E-05	T	C	0.8589	0.787	4	96491914	*UNC5C*
rs79405999	3.24432	1.81035	5.81413	7.69E-05	G	A	0.8613	0.7931	4	96493507	*UNC5C*
rs144481988	3.23067	1.80334	5.78772	8.08E-05	G	T	0.863	0.7978	4	96494118	*UNC5C*
rs75063956	3.22491	1.80348	5.76667	7.86E-05	G	C	0.8639	0.8002	4	96494298	*UNC5C*
rs17273997	0.85607	0.79191	0.925424	9.23E-05	T	G	0.9756	0.9978	5	9721047	*LOC285692*
rs4301203	0.85589	0.791646	0.925355	9.29E-05	G	A	0.964	0.9618	5	9725725	*LOC285692*
rs56903072	0.82632	0.752913	0.906881	5.84E-05	A	G	0.7915	0.7817	5	9726508	*LOC285692*
rs17333663	0.85411	0.789943	0.92348	7.56E-05	C	G	0.9522	0.9501	5	9727164	*LOC285692*
rs10062592	0.85607	0.791762	0.925607	9.61E-05	G	A	0.9525	0.9502	5	9729786	*LOC285692*
rs841137	0.80509	0.73772	0.878615	1.16E-06	A	C	0.972	0.9725	5	87072961	*CCNH*
rs12519727	1.17147	1.09606	1.25207	3.13E-06	T	C	0.9754	0.98	5	87073282	*CCNH*
rs7732500	1.17147	1.09606	1.25207	3.13E-06	G	T	0.978	0.9831	5	87074449	*CCNH*
rs1098588	0.83939	0.783783	0.89893	5.53E-07	A	G	0.9658	0.9817	5	87075328	*CCNH*
rs840850	0.80336	0.736181	0.876673	8.92E-07	G	A	0.9907	0.9918	5	87079029	*CCNH*
rs16902806	1.17332	1.0996	1.25197	1.38E-06	G	A	0.9965	0.9875	5	87081822	*CCNH*
rs710375	0.79789	0.733802	0.867579	1.26E-07	T	C	0.9984	0.9998	5	87082276	*CCNH*
rs710374	0.80756	0.744078	0.876463	3.11E-07	C	T	0.9947	0.9942	5	87082947	*CCNH*
rs710373	0.80703	0.743629	0.875827	2.80E-07	T	C	0.9967	0.9969	5	87083187	*CCNH*
rs698061	0.80551	0.738194	0.878958	1.19E-06	T	C	0.9984	0.9998	5	87085820	*CCNH*
rs698060	0.80551	0.738194	0.878958	1.19E-06	A	G	0.9985	1	5	87086376	*CCNH*
rs11740855	1.17422	1.09871	1.25491	2.18E-06	T	C	0.9586	0.9971	5	87086974	*CCNH*
rs16902819	1.1763	1.10066	1.25714	1.68E-06	G	A	0.934	0.9999	5	87089786	*CCNH*
rs698059	0.80144	0.733147	0.876086	1.11E-06	G	A	0.9348	0.9797	5	87090272	*CCNH*
rs11950745	1.17383	1.09615	1.25701	4.48E-06	C	G	0.8842	0.9133	5	87095901	*CCNH*
rs36087313	1.1681	1.0869	1.25536	2.37E-05	C	T	0.8173	0.832	5	87096545	*CCNH*
rs7722036	1.16093	1.0783	1.24988	7.46E-05	T	C	0.7606	0.7548	5	105764856	*LOC345571*
rs10064693	1.42824	1.19967	1.70035	6.18E-05	T	G	0.957	0.935	5	111810362	*FLJ11235*
rs67638655	1.43316	1.2038	1.7062	5.24E-05	G	C	0.9574	0.9363	5	111810538	*FLJ11235*
rs78669778	1.42559	1.19745	1.69719	6.75E-05	G	T	0.9642	0.9353	5	111811152	*FLJ11235*
rs1019473	1.4241	1.20044	1.68944	5.00E-05	G	A	0.9823	0.9361	5	111813496	*FLJ11235*
rs10071182	1.41888	1.19417	1.68587	6.97E-05	T	C	0.9824	0.9351	5	111813505	*FLJ11235*
rs6882162	1.41574	1.1955	1.67655	5.58E-05	A	G	0.9961	0.9424	5	111814318	*FLJ11235*
rs55979676	1.13828	1.0667	1.21467	9.29E-05	G	A	0.9995	0.9768	5	124147028	*RPL28P3*
rs187509	0.73149	0.626373	0.854243	7.81E-05	G	C	0.9606	0.9734	5	163993614	*LOC100507193*
rs250599	0.73144	0.6253	0.855584	9.24E-05	G	A	0.9556	0.9828	5	163995518	*LOC100507193*
rs165068	0.73121	0.626166	0.853869	7.60E-05	C	G	0.957	0.9877	5	163998414	*LOC100507193*
rs165069	0.73119	0.626186	0.853803	7.55E-05	A	T	0.9558	0.9895	5	163999663	*LOC100507193*
rs76000024	1.47684	1.21546	1.79444	8.74E-05	T	C	0.7231	0.7148	6	8588542	*LOC100506207*
rs78785610	1.48866	1.22088	1.81518	8.41E-05	C	G	0.7279	0.721	6	8611480	*LOC100506207*
rs2504082	0.86797	0.808757	0.931515	8.57E-05	A	G	1	0.9988	6	39851679	*DAAM2*
rs3003929	0.86797	0.808757	0.931515	8.57E-05	A	G	1	0.9984	6	39851818	*DAAM2*
rs3003932	0.86797	0.808757	0.931515	8.57E-05	G	A	0.9994	0.9976	6	39852438	*DAAM2*
rs3003933	0.86797	0.808757	0.931515	8.57E-05	C	T	0.9993	0.9973	6	39852578	*DAAM2*
rs3003934	0.86719	0.80803	0.930677	7.72E-05	A	G	0.9991	0.9971	6	39852633	*DAAM2*
rs3008806	0.86719	0.80803	0.930677	7.72E-05	T	C	0.9989	0.9969	6	39852684	*DAAM2*
rs3003935	0.86901	0.809728	0.932633	9.84E-05	G	A	0.9986	0.9961	6	39852850	*DAAM2*
rs3003936	0.86693	0.807787	0.930397	7.46E-05	A	G	0.995	0.9937	6	39852965	*DAAM2*

rs3003937	0.86719	0.80803	0.930677	7.72E-05	A	G	0.9986	0.9964	6	39853139	*DAAM2*
rs3003938	0.86719	0.80803	0.930677	7.72E-05	G	A	0.9983	0.9961	6	39853190	*DAAM2*
rs3003939	0.86719	0.80803	0.930677	7.72E-05	A	G	0.9982	0.996	6	39853364	*DAAM2*
rs3008807	0.86719	0.80803	0.930677	7.72E-05	T	G	0.998	0.9958	6	39853962	*DAAM2*
rs3003941	0.86719	0.80803	0.930677	7.72E-05	C	T	0.998	0.9958	6	39854077	*DAAM2*
rs3008808	0.86641	0.807303	0.929839	6.96E-05	T	C	0.9974	0.9958	6	39854660	*DAAM2*
rs3003943	0.86563	0.806576	0.929003	6.26E-05	C	A	0.9971	0.9959	6	39855000	*DAAM2*
rs3003944	0.86563	0.806576	0.929003	6.26E-05	C	A	0.9964	0.996	6	39855019	*DAAM2*
rs12660592	1.46812	1.21211	1.7782	8.58E-05	G	T	0.801	0.9201	6	48536972	*RBMXP1*
rs7753358	0.86059	0.799057	0.926857	7.28E-05	A	T	0.8028	0.759	6	79665210	*PHIP*
rs7742431	0.87535	0.818776	0.935829	9.40E-05	G	A	0.9997	0.9995	6	79679577	*PHIP*
rs9359359	0.87535	0.818776	0.935829	9.40E-05	C	T	0.9988	0.9998	6	79700980	*PHIP*
rs9443637	0.87535	0.818776	0.935829	9.40E-05	T	C	0.9981	0.9999	6	79714708	*PHIP*
rs9448607	0.87535	0.818776	0.935829	9.40E-05	A	G	0.9981	1	6	79715620	*PHIP*
rs495535	0.8264	0.751824	0.908364	7.76E-05	T	C	0.9342	0.9996	6	102019106	*GRIK2*
rs73531543	1.31544	1.15798	1.4943	2.50E-05	A	C	0.7896	0.7805	6	112670669	*RFPL4B*
rs9387072	1.19898	1.095	1.31284	8.82E-05	T	G	0.9673	0.9642	6	112715275	*FEM1AP3*
rs9398310	1.19898	1.095	1.31284	8.82E-05	T	A	0.9673	0.9642	6	112715276	*FEM1AP3*
rs638967	1.15844	1.07799	1.24488	6.20E-05	T	C	0.9963	0.9969	6	143052063	*HIVEP2*
rs12192768	1.16987	1.08459	1.26186	4.85E-05	A	G	0.9949	0.9967	6	143053893	*HIVEP2*
rs7349872	1.16987	1.08459	1.26186	4.85E-05	T	C	0.9946	0.997	6	143054384	*HIVEP2*
rs655648	0.8751	0.820072	0.93383	5.68E-05	C	T	0.993	0.999	6	143057185	*HIVEP2*
rs6570526	1.14647	1.07053	1.2278	9.27E-05	G	C	0.9951	0.9944	6	143058692	*HIVEP2*
rs197493	0.87354	0.818603	0.932157	4.50E-05	T	A	0.9885	0.9915	6	143060909	*HIVEP2*
rs197492	0.87078	0.814554	0.930879	4.84E-05	A	C	0.9763	0.9797	6	143061160	*HIVEP2*
rs517096	1.18213	1.08768	1.28479	8.22E-05	G	T	0.9868	0.9885	6	143062584	*HIVEP2*
rs197490	0.8736	0.818659	0.932221	4.54E-05	T	C	0.9988	0.9993	6	143062803	*HIVEP2*
rs197489	1.14895	1.07684	1.22589	2.69E-05	C	T	0.9852	0.9833	6	143063548	*HIVEP2*
rs197488	1.14436	1.07254	1.22099	4.55E-05	G	A	0.9896	0.9898	6	143064339	*HIVEP2*
rs117906471	1.39827	1.18342	1.65213	8.20E-05	C	A	0.7325	0.7377	7	18031869	*LOC100420223*
rs117985481	1.39343	1.18136	1.64357	8.20E-05	G	A	0.7303	0.7394	7	18039756	*PRPS1L1*
rs714238	0.87319	0.818386	0.93166	4.12E-05	C	T	0.9999	0.9999	7	19803925	*TMEM196*
rs12672557	0.87303	0.818181	0.931554	4.10E-05	G	A	0.9994	0.9995	7	19804237	*TMEM196*
rs1468288	0.87369	0.817451	0.933801	6.96E-05	C	G	0.9529	0.9824	7	19810580	*TMEM196*
rs79950921	0.83374	0.761043	0.913381	9.37E-05	T	C	0.988	0.9925	7	82966739	*SEMA3E*
rs80342104	0.83057	0.758153	0.909912	6.66E-05	T	C	0.9681	0.9948	7	82967129	*SEMA3E*
rs112023464	0.83057	0.758153	0.909912	6.66E-05	G	T	0.9679	0.9944	7	82967272	*SEMA3E*
rs77377734	0.83057	0.758153	0.909912	6.66E-05	G	A	0.9676	0.9941	7	82967366	*SEMA3E*
rs9690922	1.31291	1.1679	1.47593	5.14E-06	A	G	0.8297	0.9687	7	82968522	*SEMA3E*
rs147710078	0.76296	0.666288	0.87365	9.07E-05	T	C	0.8822	0.8824	7	82976315	*SEMA3E*
rs6997650	0.84102	0.778779	0.908239	1.02E-05	C	T	0.9749	0.7673	8	21322788	*GFRA2*
rs4739248	0.84737	0.784657	0.915095	2.43E-05	G	A	0.9941	0.7767	8	21323689	*GFRA2*
rs9644577	0.84822	0.785442	0.916011	2.71E-05	G	A	0.9976	0.7785	8	21324811	*GFRA2*
rs7013453	0.84822	0.785442	0.916011	2.71E-05	G	A	0.9874	0.779	8	21326288	*GFRA2*
rs7013613	0.84822	0.785442	0.916011	2.71E-05	G	A	0.9861	0.7793	8	21326379	*GFRA2*
rs10093820	1.25418	1.12186	1.40212	6.86E-05	A	G	0.905	0.8344	8	27029960	*STMN4*
rs28676517	1.25544	1.12298	1.40352	6.37E-05	A	G	0.9045	0.8302	8	27039460	*STMN4*
rs881299	0.87241	0.814516	0.934428	9.79E-05	T	C	0.9636	0.9475	8	38332249	*FGFR1*
rs56277720	0.86046	0.800426	0.924998	4.65E-05	C	T	0.9675	0.9577	8	102657443	*GRHL2*
rs56175710	0.86101	0.799558	0.927185	7.46E-05	G	A	0.9569	0.9448	8	102657510	*GRHL2*
rs2387623	0.86085	0.802275	0.923692	3.08E-05	T	A	0.9968	0.9815	8	102658409	*GRHL2*
rs4317540	0.86085	0.802275	0.923692	3.08E-05	A	C	0.9971	0.9815	8	102658532	*GRHL2*
rs2211914	0.87764	0.822737	0.936212	7.50E-05	A	G	0.9902	0.9999	8	102669667	*GRHL2*
rs2387622	0.87629	0.821465	0.934764	6.16E-05	G	A	0.995	0.9952	8	102673258	*GRHL2*
rs4734572	0.87819	0.823194	0.936869	8.28E-05	G	T	0.9996	0.9962	8	102677327	*GRHL2*
rs4734573	0.87819	0.823194	0.936869	8.28E-05	C	G	0.9998	0.9963	8	102677805	*GRHL2*
rs12549642	0.87819	0.823194	0.936869	8.28E-05	T	C	1	0.9965	8	102678146	*GRHL2*
rs3735714	0.87571	0.819529	0.935752	8.76E-05	C	T	0.9904	0.9881	8	102679322	*GRHL2*
rs4734580	0.86439	0.803975	0.92934	8.07E-05	G	A	0.9574	0.9233	8	102684412	*GRHL2*
rs7829633	0.8627	0.804058	0.925621	3.92E-05	G	A	0.9906	0.988	8	102686083	*GRHL2*
rs7829786	0.8627	0.804058	0.925621	3.92E-05	G	A	0.9902	0.9877	8	102686182	*GRHL2*
rs142765538	0.86356	0.804863	0.926547	4.43E-05	T	G	0.9908	0.9892	8	102686302	*GRHL2*

rs59320315	0.86292	0.804262	0.925856	4.05E-05	G	T	0.9939	0.9855	8	102689543	*GRHL2*
rs7386202	0.86292	0.804262	0.925856	4.05E-05	C	T	0.9872	0.9874	8	102690615	*NCALD*
rs148913427	0.86397	0.805238	0.926979	4.68E-05	T	A	0.9985	0.9968	8	102693882	*NCALD*
rs12414007	1.16924	1.08348	1.26178	5.74E-05	A	T	0.9987	0.949	10	3882089	*KLF6*
rs12415309	1.16876	1.08304	1.26127	6.00E-05	G	A	0.993	0.9527	10	3882550	*KLF6*
rs59107176	1.18282	1.0899	1.28366	5.76E-05	G	A	0.9114	0.8679	10	3882667	*KLF6*
rs12414080	1.17865	1.08802	1.27684	5.67E-05	A	T	0.9325	0.8873	10	3882668	*KLF6*
rs78424159	1.16771	1.08207	1.26014	6.62E-05	C	T	0.9994	0.962	10	3884797	*KLF6*
rs60558804	1.16771	1.08207	1.26014	6.62E-05	A	G	0.9995	0.9637	10	3885571	*KLF6*
rs61464031	1.16771	1.08207	1.26014	6.62E-05	T	C	0.9996	0.9643	10	3885746	*KLF6*
rs12411753	1.16789	1.0823	1.26025	6.42E-05	G	A	0.9999	0.966	10	3887162	*KLF6*
rs9783264	1.21261	1.10366	1.33233	5.99E-05	G	C	0.9989	0.9971	10	3920602	*KLF6*
rs729440	1.21261	1.10366	1.33233	5.99E-05	T	C	0.9991	0.9971	10	3921137	*KLF6*
rs150498816	1.2152	1.10601	1.33517	4.96E-05	A	G	0.9978	0.9972	10	3925957	*KLF6*
rs12570574	1.3954	1.18091	1.64885	9.12E-05	C	T	0.9646	0.9335	10	8665428	*LOC338591*
rs2399659	0.84965	0.786076	0.918357	4.02E-05	A	G	0.9717	0.9996	10	11238195	*CELF2*
rs1109860	0.81299	0.735282	0.898917	5.37E-05	A	G	0.994	0.9964	10	11246337	*CELF2*
rs78246937	0.78129	0.691595	0.882618	7.28E-05	A	G	0.9893	0.8546	10	31466684	*LOC100505485*
rs73248414	0.7742	0.682679	0.877999	6.69E-05	T	C	0.9511	0.8136	10	31479422	*LOC100505485*
rs7901053	0.85264	0.791294	0.918744	2.86E-05	G	A	0.9653	0.9162	10	50803542	*CHAT*
rs1917805	0.86554	0.810955	0.923797	1.39E-05	A	G	0.9991	0.9371	10	50805983	*CHAT*
rs11101179	0.83397	0.770969	0.902121	5.89E-06	T	C	0.9955	0.8962	10	50810891	*CHAT*
rs885834	0.86226	0.801849	0.927224	6.37E-05	G	A	0.9976	0.9981	10	50815512	*CHAT*
rs77595889	1.2855	1.13637	1.45421	6.56E-05	T	C	0.8854	0.9477	10	64457405	*ZNF365*
rs7129217	1.16699	1.08096	1.25988	7.74E-05	C	T	0.9984	0.8491	11	99608867	*CNTN5*
rs4438001	1.1616	1.08009	1.24926	5.45E-05	G	A	0.9507	0.8699	11	99609374	*CNTN5*
rs11821296	1.17513	1.0855	1.27216	6.70E-05	A	G	0.9031	0.9179	11	124303201	*OR8B8*
rs7315004	1.14472	1.07088	1.22364	7.10E-05	A	T	0.9796	0.972	12	31928182	*H3F3C*
rs2066920	0.84495	0.778726	0.916812	5.22E-05	T	C	0.9961	0.9749	12	102108301	*CHPT1*
rs1544922	0.84487	0.778757	0.916601	5.02E-05	T	C	0.9963	0.9974	12	102109893	*CHPT1*
rs1544920	0.84563	0.779453	0.91742	5.51E-05	G	A	0.9971	0.9983	12	102111430	*CHPT1*
rs1134165	0.84563	0.779453	0.91742	5.51E-05	A	T	0.997	0.9982	12	102112291	*CHPT1*
rs7957552	0.84563	0.779453	0.91742	5.51E-05	C	T	0.9955	0.9964	12	102118268	*CHPT1*
rs8181675	0.84563	0.779453	0.91742	5.51E-05	C	T	0.9954	0.9962	12	102120363	*CHPT1*
rs4764650	0.84563	0.779453	0.91742	5.51E-05	T	G	0.9954	0.9962	12	102121093	*CHPT1*
rs12296641	0.84554	0.77937	0.917322	5.45E-05	G	A	0.9958	0.9954	12	102124766	*SYCP3*
rs11110987	0.84498	0.778805	0.916782	5.16E-05	C	G	0.9944	0.9944	12	102129081	*SYCP3*
rs11110988	0.84498	0.778805	0.916782	5.16E-05	G	A	0.9945	0.9944	12	102129167	*SYCP3*
rs75001229	0.84387	0.77778	0.915575	4.51E-05	A	G	0.9946	0.996	12	102137156	*GNPTAB*
rs1980144	0.84387	0.77778	0.915575	4.51E-05	G	C	0.9946	0.996	12	102138708	*GNPTAB*
rs11110997	0.84347	0.777413	0.915144	4.29E-05	T	C	0.9967	0.9956	12	102147088	*GNPTAB*
rs11111001	0.84367	0.777646	0.915293	4.33E-05	C	T	0.9981	0.996	12	102151977	*GNPTAB*
rs11111007	0.84451	0.778424	0.916208	4.81E-05	T	C	0.9992	0.9967	12	102159523	*GNPTAB*
rs11111013	0.84451	0.778424	0.916208	4.81E-05	T	C	0.9993	0.9968	12	102161396	*GNPTAB*
rs11111014	0.84451	0.778424	0.916208	4.81E-05	C	T	0.9992	0.9974	12	102163513	*GNPTAB*
rs11111016	0.84442	0.77834	0.91611	4.75E-05	C	A	0.9983	0.9962	12	102166527	*GNPTAB*
rs1024587	0.84283	0.77548	0.916018	5.71E-05	T	C	1	0.9974	12	102168151	*GNPTAB*
rs55829819	0.84273	0.775396	0.915919	5.65E-05	T	G	0.9995	0.9983	12	102171476	*GNPTAB*
rs55847668	0.84273	0.775396	0.915919	5.65E-05	C	T	0.9994	0.9984	12	102171587	*GNPTAB*
rs10860784	0.8441	0.777995	0.915828	4.64E-05	A	G	0.9992	0.9994	12	102173033	*GNPTAB*
rs12229901	0.84264	0.775312	0.91582	5.59E-05	G	A	0.9994	0.9996	12	102173251	*GNPTAB*
rs58474338	0.84189	0.774621	0.915004	5.11E-05	G	T	0.9989	0.9994	12	102178556	*GNPTAB*
rs11111021	0.84189	0.774621	0.915004	5.11E-05	G	A	0.9988	0.9992	12	102178750	*GNPTAB*
rs11111022	0.84189	0.774621	0.915004	5.11E-05	C	T	0.9987	0.9992	12	102179141	*GNPTAB*
rs73163790	0.84189	0.774621	0.915004	5.11E-05	G	A	0.9984	0.9989	12	102181816	*GNPTAB*
rs58774153	0.84189	0.774621	0.915004	5.11E-05	C	T	0.9976	0.9984	12	102184333	*GNPTAB*
rs7961009	0.84189	0.774621	0.915004	5.11E-05	G	A	0.9969	0.998	12	102186064	*GNPTAB*
rs61938176	0.84198	0.774705	0.915103	5.17E-05	A	G	0.9966	0.9978	12	102187219	*GNPTAB*
rs17032014	0.84048	0.773325	0.913473	4.31E-05	T	C	0.9955	0.9962	12	102188419	*GNPTAB*
rs11111029	0.84198	0.774705	0.915103	5.17E-05	T	C	0.9945	0.9971	12	102194140	*GNPTAB*
rs12579766	0.84198	0.774705	0.915103	5.17E-05	G	A	0.9944	0.997	12	102194591	*GNPTAB*
rs4764821	0.83796	0.771055	0.910662	3.12E-05	C	T	0.9794	0.9788	12	102199329	*GNPTAB*

rs10860791	0.84119	0.773978	0.914244	4.70E-05	A	T	0.9812	0.986	12	102210221	*GNPTAB*
rs4764825	0.84271	0.77416	0.917333	7.71E-05	G	A	0.961	0.9927	12	102224108	*GNPTAB*
rs4764655	0.84197	0.773475	0.916521	7.08E-05	C	T	0.9604	0.9929	12	102224436	*GNPTAB*
rs12229323	0.84234	0.773876	0.916855	7.28E-05	T	C	0.9581	0.998	12	102227365	*GNPTAB*
rs61938212	0.84244	0.773965	0.916961	7.36E-05	C	T	0.9565	0.9996	12	102229248	*GNPTAB*
rs7966207	0.84244	0.773965	0.916961	7.36E-05	T	C	0.9561	0.9998	12	102229631	*GNPTAB*
rs113086173	0.84244	0.773965	0.916961	7.36E-05	C	T	0.9559	0.9994	12	102231400	*GNPTAB*
rs12229145	0.84031	0.771843	0.914859	6.02E-05	T	C	0.9201	0.9434	12	102232089	*GNPTAB*
rs11111051	0.83962	0.771266	0.914041	5.47E-05	C	T	0.9528	0.9794	12	102232116	*GNPTAB*
rs10860797	0.83475	0.766679	0.908869	3.16E-05	G	A	0.9369	0.96	12	102234742	*GNPTAB*
rs139987290	1.67567	1.32931	2.11229	1.25E-05	G	T	0.73	0.7464	12	103689511	*C12orf42*
rs138266248	1.44993	1.22211	1.72022	2.05E-05	T	C	0.9564	0.9414	12	103751139	*C12orf42*
rs17109366	1.15552	1.07483	1.24227	9.08E-05	A	G	0.9987	0.9354	14	25429027	*STXBP6*
rs17185460	1.15493	1.07428	1.24163	9.62E-05	T	C	0.9975	0.9376	14	25429598	*STXBP6*
rs10132944	1.15727	1.07653	1.24407	7.55E-05	C	T	0.9984	0.9581	14	25431918	*STXBP6*
rs2061433	1.24375	1.1156	1.38661	8.43E-05	A	T	0.9727	0.9623	14	54208950	*RPS3AP46*
rs8009949	1.26358	1.13164	1.41089	3.21E-05	G	A	0.9647	0.9978	14	54212167	*RPS3AP46*
rs7494596	1.26526	1.13305	1.41289	2.93E-05	G	C	0.9601	0.9868	14	54214467	*RPS3AP46*
rs8007126	1.28701	1.15048	1.43973	1.03E-05	A	T	0.9603	0.9804	14	54215660	*RPS3AP46*
rs10144968	0.77664	0.684524	0.881143	8.70E-05	T	G	0.993	0.9964	14	69150885	*RPL12P7*
rs76178561	0.74393	0.640992	0.863406	9.91E-05	C	G	0.9215	0.9294	15	27894740	*OCA2*
rs76833090	1.23767	1.11729	1.37102	4.42E-05	C	T	0.9313	0.874	15	53097320	*ONECUT1*
rs1899755	1.23541	1.11567	1.36799	4.82E-05	C	A	0.9322	0.9023	15	53102050	*ONECUT1*
rs78286545	1.23431	1.11669	1.36433	3.79E-05	C	T	0.9343	0.9189	15	53106921	*ONECUT1*
rs7168406	1.23295	1.11545	1.36283	4.16E-05	C	T	0.9326	0.9114	15	53107054	*ONECUT1*
rs76874021	1.2411	1.12275	1.37193	2.40E-05	T	C	0.9231	0.8989	15	53111247	*ONECUT1*
rs77423322	1.25293	1.13345	1.385	1.03E-05	A	T	0.9459	0.9313	15	53112139	*ONECUT1*
rs2440371	1.2503	1.13107	1.38209	1.25E-05	G	C	0.942	0.9196	15	53113112	*ONECUT1*
rs2440370	1.2503	1.13107	1.38209	1.25E-05	G	A	0.9421	0.9198	15	53113429	*ONECUT1*
rs8027820	1.24492	1.12621	1.37615	1.83E-05	C	T	0.9375	0.9275	15	53117000	*ONECUT1*
rs144399742	1.2343	1.11224	1.36975	7.42E-05	G	A	0.9621	0.9473	15	53135935	*RPSAP55*
rs61331678	1.18294	1.08904	1.28493	6.86E-05	G	C	0.8886	0.6635	15	77995949	*LOC253044*
rs12443877	0.8548	0.79044	0.924394	8.55E-05	T	C	0.8327	0.7644	16	16930411	*LOC441750*
rs4408552	1.17543	1.08384	1.27476	9.42E-05	A	G	0.9471	0.9798	16	20383531	*PDILT*
rs34532024	1.17231	1.08274	1.2693	8.85E-05	C	G	0.988	0.9853	16	20385148	*PDILT*
rs35449439	1.17231	1.08274	1.2693	8.85E-05	G	C	0.9885	0.9858	16	20385182	*PDILT*
rs35208507	1.17349	1.08382	1.27057	7.99E-05	A	G	0.9859	0.9857	16	20388929	*PDILT*
rs77924615	1.17309	1.08346	1.27014	8.27E-05	G	A	0.9783	0.9786	16	20392332	*PDILT*
rs62034975	1.17322	1.08358	1.27028	8.18E-05	G	C	0.9838	0.9819	16	20392415	*PDILT*
rs79746097	1.17322	1.08358	1.27028	8.18E-05	T	C	0.9838	0.982	16	20392810	*PDILT*
rs35747824	1.17335	1.0837	1.27042	8.08E-05	A	T	0.9839	0.9822	16	20393308	*PDILT*
rs7206790	0.81872	0.747718	0.896465	1.55E-05	C	G	0.7276	0.785	16	53797908	*FTO*
rs28429148	0.83349	0.770975	0.901077	4.68E-06	G	A	0.8226	0.8525	16	53798319	*FTO*
rs8047587	0.8428	0.780983	0.909503	1.08E-05	G	T	0.8777	0.9226	16	53798622	*FTO*
rs9937521	0.84179	0.779642	0.908882	1.07E-05	C	T	0.9423	0.8923	16	53799296	*FTO*
rs28562191	0.84194	0.779781	0.909044	1.10E-05	C	T	0.9403	0.895	16	53799303	*FTO*
rs9937053	0.84569	0.78635	0.909512	6.33E-06	G	A	0.991	0.9824	16	53799507	*FTO*
rs9937354	0.84662	0.787211	0.910508	7.27E-06	G	A	0.9986	0.9978	16	53799847	*FTO*
rs9928094	0.84662	0.787211	0.910508	7.27E-06	A	G	0.9993	0.9985	16	53799905	*FTO*
rs9930333	0.84662	0.787211	0.910508	7.27E-06	T	G	1	0.9992	16	53799977	*FTO*
rs9930397	0.84662	0.787211	0.910508	7.27E-06	T	A	1	0.999	16	53799985	*FTO*
rs9940278	0.84654	0.787137	0.910422	7.18E-06	C	T	1	0.9973	16	53800200	*FTO*
rs9939973	0.84646	0.787063	0.910336	7.10E-06	G	A	1	0.997	16	53800568	*FTO*
rs9940646	0.84646	0.787063	0.910336	7.10E-06	C	G	1	0.997	16	53800629	*FTO*
rs9940128	0.84646	0.787063	0.910336	7.10E-06	G	A	1	0.9969	16	53800754	*FTO*
rs1421085	0.82244	0.761983	0.887703	5.23E-07	T	C	1	0.9954	16	53800954	*FTO*
rs1421086	0.84569	0.78635	0.909512	6.33E-06	C	A	1	0.9968	16	53801343	*FTO*
rs9923147	0.84646	0.787063	0.910336	7.10E-06	C	T	0.993	0.991	16	53801549	*FTO*
rs9923544	0.84569	0.78635	0.909512	6.33E-06	C	T	0.9999	0.9967	16	53801985	*FTO*
rs11642015	0.82176	0.761348	0.886962	4.68E-07	C	T	0.9788	0.9707	16	53802494	*FTO*
rs1558901	0.84561	0.786276	0.909427	6.25E-06	A	T	0.9999	0.9967	16	53803187	*FTO*
rs62048402	0.82162	0.761222	0.886815	4.58E-07	G	A	0.9998	0.9956	16	53803223	*FTO*

rs1558902	0.82162	0.761222	0.886815	4.58E-07	T	A	0.9998	0.9957	16	53803574	*FTO*
rs11075985	0.84553	0.786202	0.909341	6.18E-06	C	A	0.9998	0.9968	16	53805207	*FTO*
rs56094641	0.82154	0.761148	0.886729	4.52E-07	A	G	0.9998	0.9962	16	53806453	*FTO*
rs55872725	0.82221	0.761761	0.887444	5.03E-07	C	T	0.9998	0.9968	16	53809123	*FTO*
rs1121980	0.8459	0.786545	0.909737	6.53E-06	G	A	0.9999	0.9972	16	53809247	*FTO*
rs7187250	0.82361	0.763064	0.888962	6.32E-07	C	A	0.9974	0.9971	16	53810546	*FTO*
rs7193144	0.82659	0.765825	0.892178	1.02E-06	T	C	1	0.9973	16	53810686	*FTO*
rs62033399	0.82659	0.765825	0.892178	1.02E-06	C	T	0.9998	0.9974	16	53810943	*FTO*
rs62033400	0.82576	0.765059	0.891286	8.92E-07	A	G	0.9921	0.9891	16	53811788	*FTO*
rs8063057	0.82576	0.765059	0.891286	8.92E-07	T	C	0.9995	0.9978	16	53812433	*FTO*
rs8057044	0.85171	0.790869	0.917235	2.19E-05	G	A	0.9309	0.9984	16	53812614	*FTO*
rs17817449	0.82502	0.764369	0.890482	7.92E-07	T	G	0.9998	0.9983	16	53813367	*FTO*
rs8043757	0.82494	0.764295	0.890395	7.82E-07	A	T	0.9998	0.9984	16	53813450	*FTO*
rs9972653	0.82568	0.764985	0.8912	8.80E-07	G	T	0.9997	0.9993	16	53814363	*FTO*
rs17817497	0.82562	0.764972	0.891077	8.54E-07	T	C	0.9999	0.9998	16	53815435	*FTO*
rs8050136	0.82562	0.764972	0.891077	8.54E-07	C	A	1	0.9999	16	53816275	*FTO*
rs8051591	0.82576	0.765059	0.891286	8.92E-07	A	G	0.9998	0.999	16	53816752	*FTO*
rs9935401	0.82601	0.765282	0.891546	9.26E-07	G	A	0.9996	0.9986	16	53816838	*FTO*
rs9933509	0.85108	0.791406	0.915255	1.38E-05	T	C	0.9984	0.9997	16	53818167	*FTO*
rs3751812	0.82793	0.767108	0.893565	1.23E-06	G	T	1	1	16	53818460	*FTO*
rs3751813	0.85925	0.80212	0.920441	1.55E-05	G	T	0.9607	0.991	16	53818708	*FTO*
rs3751814	0.82793	0.767108	0.893565	1.23E-06	G	A	1	0.9996	16	53818724	*FTO*
rs9931900	0.8512	0.791474	0.915438	1.42E-05	A	G	0.9983	0.9994	16	53818813	*FTO*
rs56313538	0.82801	0.767183	0.893653	1.24E-06	A	G	1	0.9992	16	53818834	*FTO*
rs9936385	0.82801	0.767183	0.893653	1.24E-06	T	C	1	0.999	16	53819169	*FTO*
rs9923233	0.82801	0.767183	0.893653	1.24E-06	G	C	0.9996	0.9988	16	53819198	*FTO*
rs9923312	0.82798	0.767112	0.893678	1.26E-06	G	A	0.9993	0.9987	16	53819367	*FTO*
rs11075988	0.82723	0.76642	0.892871	1.12E-06	G	A	0.9922	0.9901	16	53819771	*FTO*
rs11075989	0.82806	0.767187	0.893765	1.28E-06	C	T	0.9986	0.9984	16	53819877	*FTO*
rs11075990	0.82806	0.767187	0.893765	1.28E-06	A	G	0.9983	0.9982	16	53819893	*FTO*
rs11075991	0.82806	0.767187	0.893765	1.28E-06	A	T	0.998	0.9981	16	53819937	*FTO*
rs11075992	0.82814	0.767261	0.893852	1.30E-06	T	C	0.9974	0.9979	16	53820066	*FTO*
rs9926289	0.82739	0.766569	0.893045	1.15E-06	G	A	0.9968	0.9978	16	53820503	*FTO*
rs9939609	0.82739	0.766569	0.893045	1.15E-06	T	A	0.9966	0.9977	16	53820527	*FTO*
rs9937709	0.85136	0.791623	0.91561	1.46E-05	A	G	0.9951	0.9978	16	53820813	*FTO*
rs9927317	0.82646	0.765657	0.892087	1.01E-06	C	G	0.9854	0.988	16	53820996	*FTO*
rs17817712	0.82747	0.766643	0.893132	1.17E-06	A	G	0.9958	0.9975	16	53821125	*FTO*
rs7206410	0.82747	0.766643	0.893132	1.17E-06	T	C	0.9955	0.9975	16	53821297	*FTO*
rs7206629	0.85144	0.791697	0.915696	1.47E-05	T	C	0.994	0.9976	16	53821413	*FTO*
rs7202116	0.82756	0.766718	0.893218	1.18E-06	A	G	0.9948	0.9974	16	53821615	*FTO*
rs7202296	0.82764	0.766792	0.893305	1.20E-06	A	G	0.9945	0.9974	16	53821690	*FTO*
rs7201850	0.8516	0.791846	0.915869	1.51E-05	C	T	0.9934	0.9976	16	53821862	*FTO*
rs66908032	0.82772	0.766867	0.893392	1.21E-06	C	A	0.9941	0.9974	16	53822142	*FTO*
rs72803697	0.82772	0.766867	0.893392	1.21E-06	C	T	0.9914	0.9974	16	53822183	*FTO*
rs62033403	0.8278	0.766941	0.893479	1.23E-06	A	G	0.9913	0.9975	16	53822237	*FTO*
rs62033404	0.8278	0.766941	0.893479	1.23E-06	A	G	0.9912	0.9975	16	53822239	*FTO*
rs62033405	0.82788	0.767016	0.893566	1.24E-06	C	T	0.9911	0.9976	16	53822387	*FTO*
rs7206122	0.82788	0.767016	0.893566	1.24E-06	G	A	0.991	0.9976	16	53822440	*FTO*
rs7190396	0.82823	0.764499	0.897283	3.97E-06	T	G	0.8923	0.8929	16	53822502	*FTO*
rs7185735	0.82796	0.76709	0.893652	1.26E-06	A	G	0.9908	0.9978	16	53822651	*FTO*
rs79994966	0.82911	0.768156	0.894894	1.51E-06	T	C	0.9834	0.9928	16	53823727	*FTO*
rs28432761	0.84221	0.783451	0.905381	3.26E-06	T	C	0.9406	0.9789	16	53823878	*FTO*
rs11647020	0.84458	0.78571	0.90787	4.61E-06	C	T	0.9392	0.9726	16	53823990	*FTO*
rs11646715	0.8436	0.786102	0.905307	2.34E-06	G	A	0.957	0.9937	16	53824007	*FTO*
rs9941349	0.8531	0.793236	0.917476	1.87E-05	C	T	0.9907	1	16	53825488	*FTO*
rs56137030	0.83151	0.770296	0.897596	2.26E-06	G	A	0.9379	0.9448	16	53825905	*FTO*
rs28567725	0.8531	0.793236	0.917476	1.87E-05	T	C	0.9908	0.9988	16	53826028	*FTO*
rs9931494	0.85302	0.793161	0.917389	1.85E-05	C	G	0.9908	0.9983	16	53827179	*FTO*
rs10468280	0.82911	0.768156	0.894894	1.51E-06	A	G	0.9895	0.9977	16	53827479	*FTO*
rs62033408	0.82911	0.768156	0.894894	1.51E-06	A	G	0.9904	0.9972	16	53827962	*FTO*
rs17817964	0.82651	0.765659	0.892192	1.04E-06	C	T	0.9132	0.9032	16	53828066	*FTO*
rs62033413	0.83295	0.771667	0.89909	2.75E-06	C	G	0.9973	0.9856	16	53830055	*FTO*

rs9930501	0.8559	0.795843	0.920492	2.77E-05	A	G	0.9982	0.9834	16	53830452	*FTO*
rs9930506	0.8559	0.795843	0.920492	2.77E-05	A	G	0.9984	0.9833	16	53830465	*FTO*
rs9932754	0.85582	0.795769	0.920405	2.74E-05	T	C	0.9988	0.9833	16	53830491	*FTO*
rs9933040	0.85582	0.795769	0.920405	2.74E-05	T	A	0.9994	0.9832	16	53830867	*FTO*
rs9922708	0.85582	0.795769	0.920405	2.74E-05	C	T	1	0.9832	16	53831146	*FTO*
rs72805611	0.83324	0.77325	0.897892	1.71E-06	C	T	0.9999	0.9799	16	53831354	*FTO*
rs9922619	0.85582	0.795769	0.920405	2.74E-05	G	T	1	0.9831	16	53831771	*FTO*
rs7188250	0.82792	0.76554	0.895378	2.30E-06	T	C	0.9623	0.9384	16	53834607	*FTO*
rs72805612	0.82997	0.768819	0.895975	1.81E-06	G	A	0.9779	0.957	16	53834608	*FTO*
rs11075993	0.83255	0.771298	0.898659	2.59E-06	G	T	0.9976	0.9792	16	53837144	*FTO*
rs72805613	0.83247	0.771224	0.898573	2.56E-06	A	G	0.9975	0.9792	16	53837342	*FTO*
rs12149574	0.83223	0.771002	0.898315	2.47E-06	C	G	0.9944	0.9712	16	53840525	*FTO*
rs12149832	0.83484	0.773426	0.901139	3.66E-06	G	A	0.9976	0.9735	16	53842908	*FTO*
rs11649091	0.8338	0.771067	0.901632	5.24E-06	T	G	0.9533	0.941	16	53845169	*FTO*
rs61733129	1.37606	1.17186	1.61584	9.81E-05	C	T	0.9774	0.9944	16	72057421	*DHODH*
rs72787038	1.39813	1.21899	1.60358	1.66E-06	A	C	0.9832	0.9935	16	72066102	*DHODH*
rs59958340	1.38444	1.179	1.62568	7.21E-05	G	A	0.9926	0.9988	16	72071452	*DHODH*
rs76809174	1.38444	1.179	1.62568	7.21E-05	C	T	0.994	0.9994	16	72076668	*HP*
rs1424241	1.4214	1.2394	1.63013	4.90E-07	G	A	0.9988	0.9998	16	72078907	*HP*
rs5467	1.41229	1.23157	1.61952	7.75E-07	C	T	0.9874	0.9978	16	72088280	*HP*
rs56129242	1.40994	1.22952	1.61683	8.76E-07	C	G	0.9852	0.9953	16	72098260	*HPR*
rs74794641	1.39221	1.1855	1.63495	5.46E-05	G	A	0.9841	0.9939	16	72102041	*HPR*
rs6499558	1.41164	1.23101	1.61878	8.02E-07	G	C	0.9844	0.9936	16	72102813	*HPR*
rs72787058	1.41164	1.23101	1.61878	8.02E-07	C	T	0.9842	0.9935	16	72105084	*HPR*
rs8047930	1.41249	1.23175	1.61976	7.67E-07	C	T	0.984	0.993	16	72106024	*HPR*
rs11075919	1.41249	1.23175	1.61976	7.67E-07	T	C	0.9839	0.9929	16	72106906	*HPR*
rs2021171	1.38659	1.18083	1.6282	6.66E-05	G	A	0.9667	0.9745	16	72110541	*HPR*
rs1065360	1.39466	1.18771	1.63768	4.93E-05	T	C	0.9854	0.9941	16	72110781	*HPR*
rs73592060	1.39557	1.18859	1.63859	4.71E-05	C	T	0.9854	0.9941	16	72111510	*HPR*
rs55741258	1.42074	1.23894	1.62922	4.98E-07	C	T	0.9871	0.9927	16	72112480	*HPR*
rs74029810	1.3978	1.1905	1.64121	4.33E-05	C	T	0.9916	0.9949	16	72113345	*HPR*
rs74029814	1.39985	1.19224	1.64362	4.01E-05	C	T	0.9927	0.9951	16	72114715	*HPR*
rs74029815	1.39967	1.19208	1.6434	4.04E-05	A	G	0.9928	0.9951	16	72115545	*TXNL4B*
rs61258384	1.39948	1.19192	1.64318	4.07E-05	T	C	0.9931	0.9952	16	72117401	*TXNL4B*
rs60449783	1.40069	1.19296	1.6446	3.89E-05	G	A	0.9931	0.9952	16	72117522	*TXNL4B*
rs56901884	1.40069	1.19296	1.6446	3.89E-05	C	A	0.9932	0.9953	16	72117823	*TXNL4B*
rs61404039	1.40051	1.1928	1.64438	3.92E-05	G	A	0.9933	0.9953	16	72118418	*TXNL4B*
rs78310258	1.39551	1.18865	1.63836	4.68E-05	A	T	0.9962	0.9978	16	72120958	*TXNL4B*
rs78873516	1.39551	1.18865	1.63836	4.68E-05	T	G	0.9981	0.998	16	72121487	*TXNL4B*
rs8182209	1.39652	1.18952	1.63955	4.50E-05	T	A	0.9985	0.9982	16	72122315	*TXNL4B*
rs74644576	1.39634	1.18936	1.63933	4.53E-05	A	G	0.9988	0.9984	16	72122761	*TXNL4B*
rs116949987	1.39634	1.18936	1.63933	4.53E-05	C	A	0.999	0.9985	16	72123423	*TXNL4B*
rs74029840	1.39596	1.18904	1.63889	4.60E-05	G	A	0.9995	0.9989	16	72124412	*TXNL4B*
rs79680841	1.3954	1.18856	1.63823	4.70E-05	G	A	0.9996	0.9997	16	72125914	*TXNL4B*
rs17666271	1.3954	1.18856	1.63823	4.70E-05	G	A	0.9996	0.9998	16	72129681	*DHX38*
rs11554764	1.3954	1.18856	1.63823	4.70E-05	T	C	0.9997	0.9997	16	72130125	*DHX38*
rs17590101	1.3954	1.18856	1.63823	4.70E-05	T	C	0.9997	0.9994	16	72132048	*DHX38*
rs61060922	1.3954	1.18856	1.63823	4.70E-05	G	T	0.9998	0.9997	16	72136154	*DHX38*
rs55772151	1.38937	1.18343	1.63116	5.88E-05	T	G	0.9995	0.9998	16	72139667	*DHX38*
rs3815192	1.38817	1.1824	1.62975	6.15E-05	T	G	0.9997	0.9999	16	72142111	*DHX38*
rs140858411	1.39621	1.18925	1.63918	4.56E-05	C	T	0.9909	0.9985	16	72149679	*DHX38*
rs4788460	1.40142	1.21947	1.61051	1.97E-06	C	T	0.984	0.9623	16	72154509	*PMFBP1*
rs11647069	1.41228	1.22986	1.62176	9.98E-07	T	C	0.9858	0.9982	16	72155237	*PMFBP1*
rs35448862	1.38742	1.1799	1.63142	7.45E-05	G	A	0.9805	0.9986	16	72155580	*PMFBP1*
rs59049640	1.41541	1.20182	1.66695	3.14E-05	G	A	0.846	0.8581	16	72172266	*PMFBP1*
rs11648622	1.3559	1.17537	1.56416	2.96E-05	G	A	0.984	0.9533	16	72193036	*PMFBP1*
rs142907036	0.5509	0.410002	0.740218	7.63E-05	A	G	0.8726	0.8674	16	82974507	*CDH13*
rs13329933	1.15461	1.07612	1.23882	6.27E-05	C	G	0.9437	0.9307	16	87868948	*SLC7A5*
rs61527026	1.15762	1.07686	1.24445	7.29E-05	G	C	0.997	0.9318	16	87878783	*SLC7A5*
rs67310965	1.15654	1.07592	1.2432	7.98E-05	A	G	0.9988	0.9358	16	87879392	*SLC7A5*
rs67288628	1.15695	1.07623	1.24373	7.79E-05	A	G	0.999	0.9354	16	87879486	*SLC7A5*
rs997761	1.15719	1.07646	1.24399	7.60E-05	T	G	0.9993	0.9325	16	87879522	*SLC7A5*

rs997762	1.15719	1.07646	1.24399	7.60E-05	A	C	0.9984	0.9322	16	87879562	*SLC7A5*
rs68149176	1.16464	1.08331	1.25207	3.68E-05	T	C	0.9399	0.8946	16	87886490	*SLC7A5*
rs66480687	1.16464	1.08331	1.25207	3.68E-05	T	C	0.9385	0.8933	16	87886545	*SLC7A5*
rs67971539	1.16047	1.07943	1.24759	5.59E-05	A	T	0.9286	0.8826	16	87886726	*SLC7A5*
rs34212603	0.79044	0.702934	0.888833	8.54E-05	A	C	0.9998	0.9976	17	53857327	*PCTP*
rs34502292	0.79044	0.702934	0.888833	8.54E-05	T	A	0.9997	0.9976	17	53857397	*PCTP*
rs4072913	0.79217	0.704478	0.890785	9.94E-05	T	C	0.9924	0.9958	17	53890874	*PCTP*
rs35532402	0.79146	0.703842	0.889981	9.34E-05	C	T	0.9986	0.9997	17	53893595	*PCTP*
rs2060972	0.79146	0.703842	0.889981	9.34E-05	A	G	0.9994	0.9998	17	53894146	*PCTP*
rs2060973	0.79146	0.703842	0.889981	9.34E-05	G	C	0.9968	0.9967	17	53895097	*PCTP*
rs2060974	0.79146	0.703842	0.889981	9.34E-05	A	G	0.9967	0.9964	17	53895117	*PCTP*
rs12944648	0.79146	0.703842	0.889981	9.34E-05	A	G	0.9966	0.9968	17	53895623	*PCTP*
rs9912189	0.79146	0.703842	0.889981	9.34E-05	T	C	0.9939	0.9973	17	53896995	*PCTP*
rs7225154	0.79146	0.703842	0.889981	9.34E-05	C	T	0.9908	0.9974	17	53897893	*PCTP*
rs34819927	0.79146	0.703842	0.889981	9.34E-05	G	T	0.9908	0.9974	17	53898015	*PCTP*
rs1454117	0.79146	0.703842	0.889981	9.34E-05	C	T	0.9906	0.9975	17	53899899	*PCTP*
rs1454116	0.79146	0.703842	0.889981	9.34E-05	T	C	0.9905	0.9975	17	53899961	*PCTP*
rs1454115	0.79146	0.703842	0.889981	9.34E-05	C	T	0.9905	0.9975	17	53899965	*PCTP*
rs1454114	0.79146	0.703842	0.889981	9.34E-05	T	C	0.9904	0.9998	17	53900177	*PCTP*
rs2290432	0.79115	0.703569	0.889636	9.09E-05	G	A	0.9903	0.9954	17	53900679	*PCTP*
rs9915597	0.79115	0.703569	0.889636	9.09E-05	A	T	0.9897	0.995	17	53901671	*PCTP*
rs117508892	0.69961	0.587816	0.832655	5.78E-05	C	G	0.8842	0.8829	17	59246512	*BCAS3*
rs139802795	0.69827	0.586693	0.831064	5.27E-05	G	A	0.886	0.8841	17	59249912	*BCAS3*
rs78156806	0.69766	0.58615	0.83038	5.08E-05	G	T	0.8872	0.8608	17	59266048	*BCAS3*
rs79554841	0.69496	0.582884	0.828576	5.00E-05	A	G	0.8841	0.8602	17	59270731	*BCAS3*
rs117462610	0.69462	0.582568	0.828213	4.91E-05	A	T	0.7872	0.8084	17	59279063	*BCAS3*
rs9914222	0.82189	0.746851	0.904472	5.94E-05	G	T	0.9231	0.7825	17	74534650	*PRCD*
rs2289607	0.83047	0.757602	0.910338	7.34E-05	G	T	0.9955	0.8602	17	74536762	*PRCD*
rs895157	0.79382	0.71309	0.883682	2.44E-05	A	C	0.8665	0.9165	17	74540917	*PRCD*
rs4648339	0.82886	0.755278	0.909618	7.59E-05	C	T	0.9967	0.9982	17	74541137	*PRCD*
rs5742903	0.81986	0.744346	0.903032	5.60E-05	T	C	0.9606	0.9641	17	74541171	*PRCD*
rs895156	0.82961	0.755958	0.910437	8.21E-05	C	A	1	0.9998	17	74541883	*PRCD*
rs4648340	0.82928	0.755656	0.910073	7.93E-05	C	T	0.9903	0.9854	17	74542217	*PRCD*
rs75246883	0.82961	0.755958	0.910437	8.21E-05	T	C	1	0.9997	17	74542335	*PRCD*
rs35455505	0.82961	0.755958	0.910437	8.21E-05	A	G	0.9998	0.9996	17	74542336	*PRCD*
rs11657534	0.82961	0.755958	0.910437	8.21E-05	A	G	0.9998	0.9996	17	74542513	*PRCD*
rs12944601	0.82749	0.752669	0.909739	8.99E-05	A	C	0.982	0.9831	17	74542628	*PRCD*
rs4648341	0.82961	0.755958	0.910437	8.21E-05	A	G	0.9997	0.9995	17	74543230	*PRCD*
rs12936288	0.82961	0.755958	0.910437	8.21E-05	C	G	0.9997	0.999	17	74543679	*PRCD*
rs55735645	0.82961	0.755958	0.910437	8.21E-05	G	T	0.9997	0.999	17	74543881	*PRCD*
rs56024043	0.82961	0.755958	0.910437	8.21E-05	C	T	0.9998	0.9992	17	74544238	*PRCD*
rs4648342	0.82961	0.755958	0.910437	8.21E-05	G	A	0.9999	0.9992	17	74545299	*PRCD*
rs7219889	0.82961	0.755958	0.910437	8.21E-05	T	C	0.9999	0.9996	17	74545592	*PRCD*
rs4346230	0.8263	0.751454	0.908606	8.20E-05	C	T	0.9972	0.9681	17	74546560	*PRCD*
rs752049	0.82961	0.755958	0.910437	8.21E-05	C	T	0.9999	0.9997	17	74546939	*PRCD*
rs12949102	0.82843	0.753625	0.91066	9.69E-05	C	T	0.9892	0.9987	17	74548115	*LOC100507246*
rs12952443	0.8076	0.725316	0.89921	9.71E-05	G	A	0.8523	0.879	17	74552376	*LOC100507246*
rs75038521	1.2811	1.13557	1.44529	5.67E-05	G	C	0.8828	0.9123	18	12975436	*SEH1L*
rs9958986	0.86129	0.804349	0.922259	1.88E-05	C	T	0.8934	0.8755	18	12995129	*CEP192*
rs12605081	1.18037	1.09467	1.27279	1.62E-05	G	A	1	0.9981	18	12999964	*CEP192*
rs7234163	0.86429	0.810221	0.921969	9.65E-06	C	T	1	0.9944	18	13000975	*CEP192*
rs4499304	0.86429	0.810221	0.921969	9.65E-06	G	C	1	0.9946	18	13001512	*CEP192*
rs76747169	1.27805	1.13318	1.44143	6.42E-05	C	T	0.8893	0.9404	18	13008741	*CEP192*
rs58871601	0.86413	0.810132	0.921728	9.18E-06	C	G	0.9993	0.9996	18	13010238	*CEP192*
rs11661322	0.86413	0.810132	0.921728	9.18E-06	T	A	0.9991	0.9997	18	13012294	*CEP192*
rs6505783	0.84274	0.783789	0.90612	3.76E-06	C	T	0.992	0.9979	18	13187952	*C18orf1*
rs6505784	0.84274	0.783789	0.90612	3.76E-06	G	A	0.9922	0.9982	18	13188031	*C18orf1*
rs17662894	0.84291	0.783945	0.906301	3.85E-06	G	A	0.9958	0.9996	18	13189100	*C18orf1*
rs2027756	0.84299	0.784024	0.906391	3.90E-06	C	T	0.9999	0.9996	18	13190107	*C18orf1*
rs12969041	0.84299	0.784024	0.906391	3.90E-06	C	T	0.9996	0.9998	18	13191184	*C18orf1*
rs4797735	0.8435	0.784494	0.906935	4.22E-06	G	T	0.9824	0.9906	18	13193492	*C18orf1*
rs7243859	0.85062	0.791323	0.914355	1.14E-05	A	G	0.7685	0.8169	18	13194566	*C18orf1*

rs12458580	0.85165	0.792129	0.915647	1.40E-05	G	A	0.9918	0.995	18	13198053	*C18orf1*
rs7343060	0.84813	0.788899	0.911797	8.19E-06	C	G	0.9442	0.984	18	13203603	*C18orf1*
rs12454589	0.84794	0.787209	0.91335	1.36E-05	C	T	0.9006	0.9511	18	13211703	*C18orf1*
rs1540043	0.8758	0.819254	0.936251	9.85E-05	G	T	1	1	18	33691313	*SLC39A6*
rs62101454	0.87565	0.819061	0.936154	9.80E-05	C	T	0.9821	0.9788	18	33691630	*SLC39A6*
rs8092264	0.87263	0.816284	0.932857	6.31E-05	A	G	0.9974	0.9986	18	33700914	*SLC39A6*
rs8093827	0.87185	0.815554	0.932023	5.64E-05	G	T	0.999	1	18	33701451	*SLC39A6*
rs3737467	0.87185	0.815554	0.932023	5.64E-05	G	C	0.999	1	18	33701866	*SLC39A6*
rs62111294	1.20252	1.1011	1.31328	4.09E-05	C	T	0.8737	0.7222	19	46442213	*NOVA2*
rs11672738	1.16961	1.08497	1.26086	4.36E-05	C	T	0.9981	0.7268	19	46451214	*NOVA2*
rs56361150	1.16904	1.08268	1.26229	6.64E-05	G	T	0.988	0.7514	19	46453624	*NOVA2*
rs11672647	1.17011	1.08367	1.26344	6.02E-05	G	T	0.9804	0.7532	19	46456764	*NOVA2*
rs4810812	0.85786	0.794359	0.926434	9.33E-05	A	G	0.782	0.992	20	47168421	*LOC100506069*
rs6066758	0.8582	0.794745	0.926717	9.54E-05	T	C	0.7833	0.9947	20	47169044	*LOC100506069*
rs976864	0.8582	0.794745	0.926717	9.54E-05	A	C	0.7837	0.9954	20	47170013	*LOC100506069*
rs1883888	0.85745	0.794054	0.925911	8.71E-05	G	A	0.7844	0.999	20	47171338	*PREX1*
rs2869403	0.85734	0.793951	0.925791	8.59E-05	G	A	0.7855	0.9981	20	47172410	*PREX1*
rs981210	0.85798	0.79454	0.926478	9.29E-05	T	C	0.7903	0.9967	20	47174186	*PREX1*
rs1358721	0.85731	0.793854	0.925845	8.72E-05	C	T	0.8058	0.9975	20	47175674	*PREX1*
rs6063273	0.85657	0.793161	0.925037	7.95E-05	C	T	0.802	0.9987	20	47176120	*PREX1*
rs2057087	0.85455	0.791368	0.922779	6.06E-05	G	T	0.7962	0.9999	20	47177768	*PREX1*
rs6063274	0.85679	0.793438	0.925193	8.02E-05	A	G	0.8151	0.9992	20	47178270	*PREX1*
rs910195	0.8553	0.792057	0.923583	6.65E-05	G	T	0.8073	0.9993	20	47178735	*PREX1*
rs910193	0.85767	0.794259	0.92615	8.95E-05	G	A	0.8146	0.9996	20	47179630	*PREX1*
rs878198	0.85767	0.794259	0.92615	8.95E-05	A	C	0.815	0.9999	20	47179653	*PREX1*
rs4810816	0.85767	0.794259	0.92615	8.95E-05	G	T	0.8158	0.9994	20	47180193	*PREX1*
rs2869404	0.85828	0.79609	0.925319	6.82E-05	A	G	0.8263	0.9987	20	47180728	*PREX1*
rs7263262	0.85828	0.79609	0.925319	6.82E-05	A	G	0.8267	0.9985	20	47181636	*PREX1*
rs910204	0.85752	0.795392	0.924509	6.19E-05	C	A	0.824	0.9984	20	47183010	*PREX1*
rs910202	0.85763	0.795491	0.924624	6.28E-05	C	G	0.8245	0.9981	20	47183061	*PREX1*
rs910201	0.86008	0.797699	0.927347	8.73E-05	A	G	0.8467	0.9747	20	47183213	*PREX1*
rs910200	0.85989	0.797585	0.927057	8.37E-05	A	C	0.8522	0.9976	20	47183864	*PREX1*
rs6125379	0.85913	0.796886	0.926245	7.61E-05	G	C	0.864	0.9953	20	47184280	*PREX1*
rs6019284	0.85985	0.798779	0.925599	5.90E-05	C	T	0.8916	0.9892	20	47184879	*PREX1*
rs1321011	0.85985	0.798779	0.925599	5.90E-05	A	G	0.895	0.9886	20	47185401	*PREX1*
rs1852970	0.85934	0.798242	0.92512	5.62E-05	C	T	0.8771	0.9709	20	47185684	*PREX1*
rs6125380	0.86128	0.801407	0.925615	4.85E-05	G	A	0.9381	0.9635	20	47186032	*PREX1*
rs6019285	0.86185	0.801944	0.926235	5.24E-05	A	G	0.9388	0.9606	20	47186044	*PREX1*
rs4810818	0.86567	0.806826	0.928807	5.91E-05	C	T	0.975	0.9877	20	47186585	*PREX1*
rs6012480	0.86577	0.806976	0.928851	5.90E-05	G	A	0.9759	0.9939	20	47187085	*PREX1*
rs6019287	0.86645	0.807608	0.929579	6.47E-05	C	T	0.9822	0.9944	20	47187307	*PREX1*
rs4810819	0.86818	0.809225	0.93144	8.17E-05	G	A	0.998	0.9996	20	47189231	*PREX1*
rs4810820	0.86818	0.809225	0.93144	8.17E-05	T	C	0.9986	0.9999	20	47189484	*PREX1*
rs6019291	0.86818	0.809225	0.93144	8.17E-05	A	G	0.9908	0.9994	20	47191233	*PREX1*
rs6019292	0.86818	0.809225	0.93144	8.17E-05	G	A	0.994	0.9998	20	47191323	*PREX1*
rs6012481	0.86741	0.808504	0.93061	7.36E-05	C	T	0.9877	0.9996	20	47191335	*PREX1*
rs6063276	0.86818	0.809225	0.93144	8.17E-05	G	T	0.9929	0.9997	20	47191787	*PREX1*
rs4809705	0.86519	0.806431	0.928224	5.44E-05	G	A	0.9612	0.9989	20	47192456	*PREX1*
rs4810821	0.86409	0.804144	0.928497	6.82E-05	T	C	0.9306	0.9996	20	47193029	*PREX1*
rs4810822	0.86409	0.804144	0.928497	6.82E-05	T	G	0.9302	0.9998	20	47193042	*PREX1*
rs4810823	0.86409	0.804144	0.928497	6.82E-05	G	A	0.9297	0.9992	20	47193275	*PREX1*
rs6090874	0.85637	0.79536	0.922055	3.92E-05	G	A	0.8671	0.9004	20	47195146	*PREX1*
rs6122700	0.85289	0.792069	0.918372	2.49E-05	C	T	0.8466	0.888	20	47196231	*PREX1*
rs6019298	0.85361	0.792683	0.919213	2.79E-05	C	G	0.8358	0.8618	20	47197336	*PREX1*
rs3934837	0.85041	0.787069	0.918846	4.08E-05	A	C	0.7084	0.7835	20	47198132	*PREX1*
rs1006209	0.85405	0.791516	0.921529	4.78E-05	T	C	0.9983	0.9949	22	26319391	*MYO18B*
rs2301504	0.85473	0.792142	0.922259	5.21E-05	T	C	0.9987	0.9954	22	26320015	*MYO18B*
rs5761319	0.853	0.790385	0.920567	4.35E-05	A	T	0.9932	0.9623	22	26322953	*MYO18B*
rs2227236	0.85002	0.786216	0.919008	4.48E-05	G	C	0.9505	0.9325	22	26326696	*MYO18B*
rs5769708	0.86872	0.810553	0.931052	6.88E-05	C	A	0.9974	0.8468	22	49365633	*LOC100128946*
