# Supplementary material for: A variant within the FTO confers susceptibility to diabetic nephropathy in Japanese patients with type 2 diabetes
Source: PLoS One. 2018 Dec 19;13(12):e0208654. doi: 10.1371/journal.pone.0208654 (PMC6300288; doi:10.1371/journal.pone.0208654)
Supplement: S2 Table — (PDF) [file pone.0208654.s005.pdf]

S2 Table: Candidate SNP loci for overt diabetic nephropathy in the discovery stage

SNPID	chr	position GRCh37.p13	Nearest gene	EA/ Non-EA	Stage1 set1 Beta	SE	P-value	Stage1 set2 Beta	SE	P-value	Stage2 Beta	SE	P-value	Joint analysis P-value
rs56094641	16	53806453	FTO	A/G	-0.199	0.041	0.000001185	-0.174	0.125	0.1635	-0.23793689	0.066899	0.0004	7.743E-10
rs895157	17	74540917	PRCD	T/G	-0.243	0.058	0.00002846	-0.133	0.165	0.4222	-0.29708212	0.087666	0.0007	7.695E-08
rs10144968	14	69150885	RAD51B	T/G	-0.242	0.068	0.0003747	-0.347	0.201	0.08475	-0.45630913	0.118349	0.0001	1.223E-07
rs13306536	1	53723222	LRP8	G/A	-0.321	0.066	0.000001387	0.14	0.206	0.4964	-0.30076665	0.114438	0.0086	2.701E-07
rs7544082	1	48203990	TRABD2B	C/A	-0.163	0.039	0.0000322	-0.137	0.099	0.1643	-0.15241083	0.058089	0.0087	3.083E-07
rs11101179	10	50810891	CHAT	T/C	-0.199	0.042	0.000002231	-0.004	0.134	0.9767	-0.15922174	0.068996	0.021	3.848E-07
rs710375	5	87082276	CCNH	T/C	-0.217	0.045	0.00000128	-0.306	0.136	0.02445	-0.07356953	0.073144	0.3145	3.961E-07
rs62111294	19	46442213	NOVA2	C/T	0.173	0.047	0.0002279	0.307	0.154	0.04571	0.208986631	0.072497	0.0039	5.565E-07
rs4858673	3	25008633	CFL1P7	G/C	0.292	0.081	0.0003342	0.363	0.222	0.103	0.439813166	0.142038	0.002	7.755E-07
rs190929035	3	150968079	MED12L	T/C	-0.699	0.174	0.00005807	-0.666	0.583	0.253	-0.6719474	0.25801	0.0092	8.609E-07
rs6729523	2	80750869	CTNNA2	A/G	-0.213	0.05	0.00001787	-0.045	0.162	0.7812	-0.20583685	0.082218	0.0123	0.000001246
rs77423322	15	53112139	ONECUT1	A/T	0.232	0.054	0.00001924	0.169	0.159	0.2907	0.168743902	0.086615	0.0514	0.000001685
rs9855025	3	5434207	MRPS35P1	T/C	-0.176	0.048	0.0002703	-0.386	0.157	0.01403	-0.17963057	0.080138	0.025	0.000001748
rs11661322	18	13012294	CEP192	T/A	-0.142	0.035	0.0000487	-0.177	0.097	0.06923	-0.10733556	0.058131	0.0648	0.000001846
rs9398310	6	112715276	FEM1AP3	T/A	0.211	0.049	0.00001991	-0.063	0.141	0.656	0.212154828	0.077608	0.0063	0.000001864
rs6505783	18	13187952	C18orf1	C/T	-0.156	0.039	0.00006537	-0.307	0.117	0.00887	-0.0930835	0.063708	0.144	0.000002214
rs75038521	18	12975436	SEH1L	G/C	0.273	0.066	0.00003368	0.08	0.17	0.6388	0.238240629	0.099086	0.0162	0.000002748
rs6122700	20	47196231	PREX1	C/T	-0.185	0.04	0.000004112	0.051	0.114	0.6527	-0.1275447	0.061738	0.0388	0.000002945
rs75851138	3	10223370	IRAK2	C/T	0.249	0.062	0.0000617	0.12	0.178	0.5006	0.230430288	0.097506	0.0181	0.00000319
rs1019473	5	111813496	FLJ11235	G/A	0.359	0.094	0.000144	0.32	0.233	0.1696	0.320330587	0.141441	0.0235	0.00000347
rs12570574	10	8665428	LOC338591	C/T	0.364	0.09	0.00005001	0.07	0.263	0.7911	0.331080488	0.135643	0.0147	0.000003995
rs197489	6	143063548	HIVEP2	C/T	0.153	0.035	0.00001697	0.021	0.101	0.8335	0.116151999	0.060746	0.0559	0.00000419
rs6661844	1	64870696	CACHD1	G/T	0.204	0.049	0.00003588	0.138	0.146	0.344	0.134441532	0.079678	0.0915	0.000006202
rs1897810	4	96016055	BMPR1B	A/G	0.167	0.036	0.000004078	0.059	0.104	0.5673	0.066242894	0.058248	0.2554	0.000006209
rs10132944	14	25431918	STXBP6	C/T	0.171	0.039	0.0000138	-0.067	0.114	0.5575	0.139764214	0.064404	0.03	0.000006381
rs59085380	1	203208785	CHIT1	T/C	0.178	0.037	0.000001984	0.035	0.106	0.7398	0.054264132	0.059	0.3577	0.000007801
rs8007126	14	54215660	RPS3AP46	A/T	0.213	0.061	0.000469	0.54	0.165	0.001082	0.128315926	0.09752	0.1882	0.00000784
rs16851159	1	203207621	CHIT1	A/G	0.178	0.037	0.000001978	0.035	0.106	0.742	0.050104169	0.059163	0.3971	0.000009188
rs3737467	18	33701866	SLC39A6	G/C	-0.116	0.036	0.00126	-0.317	0.105	0.002515	-0.10615641	0.059074	0.0723	0.00001152
rs76671526	4	96483630	UNC5C	A/G	1.328	0.324	0.00004275	0.238	1.095	0.8277	0.796173748	0.400475	0.0468	0.00001227
rs1323826	1	58232509	DAB1	A/G	-0.136	0.036	0.0001969	-0.143	0.112	0.2026	-0.10697978	0.059994	0.0746	0.00001382
rs142907036	16	82974507	CDH13	A/G	-0.636	0.159	0.00006405	-0.244	0.473	0.6055	-0.46095033	0.24849	0.0636	0.00001397
rs34212603	17	53857327	PCTP	A/C	-0.248	0.063	0.00007464	-0.116	0.192	0.5459	-0.18189989	0.104022	0.0803	0.00001892
rs5769708	22	49365633	LOC100128946	C/A	-0.168	0.037	0.00000595	0.146	0.12	0.2243	-0.10098949	0.06077	0.0965	0.00001901

rs12414007	10	3882089	KLF6	A/T	0.139	0.041	0.0007238	0.31	0.122	0.01128	0.102344345	0.067529	0.1296	0.00002207
rs10093820	8	27029960	STMN4	A/G	0.238	0.06	0.00008065	0.124	0.179	0.4881	0.143060775	0.089063	0.1082	0.00002445
rs2066920	12	102108301	CHPT1	T/C	-0.179	0.044	0.00005532	-0.078	0.129	0.5472	-0.10342057	0.07294	0.1562	0.00002484
rs117650098	4	95549420	PDLIM5	G/T	-0.488	0.133	0.0002365	-0.893	0.51	0.08023	-0.32406498	0.212138	0.1266	0.00002604
rs3008808	6	39854660	DAAM2	T/C	-0.149	0.038	0.00009687	-0.093	0.114	0.4136	-0.09276097	0.061926	0.1341	0.00002773
rs73248414	10	31479422	LOC100505485	T/C	-0.228	0.067	0.0007222	-0.568	0.224	0.01126	-0.15806113	0.106087	0.1362	0.00002885
rs12660592	6	48536972	RBMXP1	G/T	0.358	0.106	0.0007548	0.532	0.253	0.03586	0.256381675	0.163261	0.1163	0.00002962
rs9690922	7	82968522	SEMA3E	A/G	0.254	0.064	0.00006532	0.395	0.166	0.01734	0.05929871	0.088341	0.5021	0.00003282
rs78500739	4	1169631	SPON2	G/A	0.254	0.073	0.0004999	0.453	0.2	0.02357	0.160636726	0.132131	0.2241	0.00003318
rs35747824	16	20393308	PDILT	A/T	0.135	0.043	0.001671	0.36	0.122	0.003182	0.101279032	0.069838	0.147	0.00003518
rs4764821	12	102199329	GNPTAB	C/T	-0.189	0.045	0.00002237	-0.078	0.128	0.5408	-0.07751174	0.0727	0.2863	0.00003568
rs17163886	1	115956697	NGF	A/C	-0.18	0.047	0.0001523	-0.287	0.16	0.07273	-0.07649582	0.074982	0.3076	0.00003992
rs2387623	8	102658409	GRHL2	T/A	-0.134	0.038	0.0004411	-0.285	0.111	0.01045	-0.05934844	0.063322	0.3486	0.00004367
rs11821296	11	124303201	OR8B8	A/G	0.169	0.043	0.00007319	0.102	0.12	0.3944	0.083351126	0.064303	0.1949	0.00004817
rs117906471	7	18031869	LOC100420223	C/A	0.325	0.09	0.0002911	0.422	0.262	0.1067	0.167463865	0.114553	0.1438	0.00005502
rs4438001	11	99609374	CNTN5	G/A	0.137	0.039	0.000499	0.273	0.121	0.0243	0.060932614	0.064981	0.3484	0.0000721
rs17333663	5	9727164	LOC285692	C/G	-0.165	0.042	0.0001004	-0.092	0.126	0.467	-0.06479133	0.069484	0.3511	0.00009728
rs2007556	2	26532491	GPR113	T/G	0.207	0.049	0.00002166	0.112	0.143	0.433	0.030041616	0.080618	0.7094	0.0001087
rs118080383	2	62784619	RSL24D1P2	G/A	-0.207	0.058	0.0003926	-0.48	0.19	0.01163	0.048979027	0.09542	0.6077	0.0001197
rs76178561	15	27894740	OCA2	C/G	-0.296	0.08	0.0002089	-0.294	0.243	0.2277	-0.11444433	0.130002	0.3787	0.0001418
rs9359359	6	79700980	PHIP	C/T	-0.12	0.036	0.0009972	-0.247	0.106	0.01937	-0.04850861	0.059174	0.4124	0.0001486
rs13000148	2	26526763	GPR113	C/T	0.219	0.052	0.00002678	0.124	0.151	0.4096	0.024205332	0.081358	0.7661	0.0001502
rs117462610	17	59279063	BCAS3	A/T	-0.35	0.094	0.0001939	-0.513	0.302	0.08995	-0.21978808	0.152789	0.1503	0.000194
rs881299	8	38332249	FGFR1	T/C	-0.143	0.037	0.0001333	-0.08	0.109	0.4621	-0.04100924	0.05926	0.4889	0.0002109
rs77595889	10	64457405	ZNF365	T/C	0.219	0.067	0.001138	0.491	0.183	0.007245	0.059081749	0.094194	0.5305	0.0002446
rs11119680	1	211309203	KCNH1	G/C	0.155	0.044	0.0003833	0.338	0.131	0.01012	0.077390179	0.069496	0.2655	0.000383
rs2399659	10	11238195	CELF2	A/G	-0.161	0.042	0.0001178	-0.179	0.121	0.1378	0.004503648	0.069162	0.9481	0.0004163
rs13374832	1	201400917	TNNI1	G/A	0.24	0.059	0.0000478	0.151	0.17	0.3746	-0.02283944	0.09012	0.7999	0.0007178
rs165069	5	163999663	LOC100507193	A/T	-0.323	0.083	0.0001085	-0.215	0.261	0.4104	0.055382091	0.14058	0.6936	0.001128
rs66480687	16	87886545	SLC7A5	T/C	0.175	0.039	0.000008675	-0.044	0.115	0.7025	-0.1157649	0.06289	0.0657	0.008636
rs77746757	2	1788730	MYT1L	C/G	0.219	0.059	0.0001918	0.197	0.165	0.2325	-0.17783147	0.09745	0.068	0.01307
