# Supplementary material for: A variant within the FTO confers susceptibility to diabetic nephropathy in Japanese patients with type 2 diabetes
Source: PLoS One. 2018 Dec 19;13(12):e0208654. doi: 10.1371/journal.pone.0208654 (PMC6300288; doi:10.1371/journal.pone.0208654)
Supplement: S3 Table — (PDF) [file pone.0208654.s006.pdf]

S3 Table. Effects of the adjustment for age, sex and BMI on association of the *FTO* locus with susceptibility to diabetic nephropathy.

SNP	Alleles		Sample group	P -value	
	Effect	Non-effect		Unadjusted	Adjusted[a]
rs9936385	G	A	Stage-1 set1	0.0003	0.0008
rs9936385	G	A	Stage-1 set2	0.0171	0.007
rs56094641	G	A	Stage-2	0.0045	0.0029
**Meta analysis (Combined Stage-1 set-1, set-2 and Stage-2)**				3.22×10^{-7}	3.46×10^{-7}

a. Adjusted for age, sex and log-transformed BMI.
