# Supplementary material for: A variant within the FTO confers susceptibility to diabetic nephropathy in Japanese patients with type 2 diabetes
Source: PLoS One. 2018 Dec 19;13(12):e0208654. doi: 10.1371/journal.pone.0208654 (PMC6300288; doi:10.1371/journal.pone.0208654)
Supplement: S4 Table — (PDF) [file pone.0208654.s007.pdf]

S4 Table. Association of previously reported loci with overt diabetic nephropathy in the stage 1 analysis

Study	Ethnicity	Trait	Diabetes type	SNP ID	*Neaest Gene*	OR in the original study	P value in the original study	OR for the same SNP in this study	P value for the same SNP in this study	Lead SNP in this study	OR for the lead SNP	P value for the lead SNP	Neaest Gene for the lead SNP	Linkage disequilibrium coefficient (R square) between the reported SNPs and the lead SNPs in this study
Tanaka *et al.*[1]	Japanese	DN	T2D	rs11643718	*SLC12A3*	2.53	2.0×10^{-5}	1.05	4.71×10^{-1}	rs118121751	1.35	2.95 × 10-2	*SLC12A3*	0.0025
Shimazaki *et al.*[2]	Japanese	DN	T2D	rs741301	*ELMO1*	2.67	8.00×10^{-6}	0.96	2.88×10^{-1}	rs77978662	0.63	2.38 × 10-3	*ELMO1*	0.0327
Hanson *et al.*[3]	Native American (Pima Indian)	ESRD	T2D	rs2648875	*PVT1*	2.97	1.80×10^{-6}	1.06	7.78×10^{-2}	rs143059315	0.74	4.72 × 10-3	*PVT1*	0.0125
Pezzolesi *et al.*[4]	European: GoKinD,DCCT/EDIC	DN	T1D	rs39075	*CHN2*	1.43	6.50×10^{-7}	1	9.38×10^{-1}	rs13231155	1.43	1.60 × 10-2	*CHN2*	0.0121
Pezzolesi *et al.*[4]	European: GoKinD,DCCT/EDIC	DN	T1D	rs1888747	*FRMD3*	1.45	6.30×10^{-7}	1.04	3.00×10^{-1}	rs117351624	0.83	1.13 × 10-1	*FRMD3*	0.1392
Pezzolesi *et al.*[4]	European: GoKinD,DCCT/EDIC	DN	T1D	rs451041	*CARS*	1.36	3.10×10^{-6}	0.99	7.72×10^{-1}	rs146277534	0.72	5.16 × 10-2	*CARS*	0.0045
Pezzolesi *et al.*[4,5]	European: GoKinD,DCCT/EDIC, etc.	DN	T1D,T2D	rs1411766	*MYO16-IRS2*	1.41	1.80×10^{-6}	1.08	1.73×10^{-1}	rs12427608	1.24	1.97 × 10-2	*IRS2*	0.1877
Craig *et al.*[6]	European	ESRD	T1D	rs1749824	*ZMIZ1*	1.47	8.10×10^{-5}	1.02	5.25×10^{-1}	rs780159	1.12	6.00 × 10-2	*ZMIZ1*	0.2453
Craig *et al.*[6]	European	ESRD	T1D	rs9298190	*MSC*	1.56	1.60×10^{-5}	1.04	2.07×10^{-1}	rs16937875	0.89	1.59 × 10-2	*LOC100132891*	0.0679
Maeda *et al.*[7]	Japanese	DN	T2D	rs2268388	*ACACB*	1.61	5.35×10^{-8}	1.07	1.86×10^{-1}	rs17848826	1.25	2.45 × 10-3	*ACACB*	0.0194
McDonough *et al.*[8]	African American	DN	T2D	rs7769051	*RPS12*	1.28	2.20×10^{-6}	0.93	1.56×10^{-1}	rs73772989	0.79	1.78 × 10-2	*HMGB1P13*	0.0458
McDonough *et al.*[8]	African American	DN	T2D	rs6930576	*SASH1*	1.31	7.04×10^{-7}	1.03	5.97×10^{-1}	rs35120946	1.15	7.47 × 10-3	*SASH1*	0.0107
McDonough *et al.*[8]	African American	DN	T2D	rs773506	*AUH*	1.32	6.45×10^{-6}	0.97	3.59×10^{-1}	rs10991837	1.1	7.38 × 10-3	*AUH*	0.1553
McDonough *et al.*[8]	African American	DN	T2D	rs2358944	*MSRB3-HMGA2*	1.33	3.54×10^{-6}	0.97	4.50×10^{-1}	rs11175888	1.07	6.69 × 10-2	*RPSAP52*	0.3634
McDonough *et al.*[8]	African American	DN	T2D	rs2106294	*LIMK2*	1.75	4.11×10^{-6}	1.11	5.81×10^{-2}	rs182468383	1.76	4.06 × 10-3	*RNF185*	*
Sandholm *et al.*[9]	European: GENIE(UK-ROI,FinnDiane,GoKinUS)+9 follow-up studies	DN	T1D	rs12437854 (rs17709344)	*RGMA -MCTP2*	1.29	2.00×10^{-9}	0.97	4.00×10^{-1} (5.65×10^{-1})	rs75308150	1.14	6.42 × 10-4	*SEPHS1P2*	0.0243
Sandholm *et al.*[9]	European: GENIE(UK-ROI,FinnDiane,GoKinUS)+9 follow-up studies	DN	T1D	rs7583877	*AFF3*	1.29	1.20×10^{-8}	1.07	4.70×10^{-2}	rs17023082	0.92	1.57 × 10-2	*AFF3*	0.3836
Sandholm *et al.*[9]	European: GENIE(UK-ROI,FinnDiane,GoKinUS)+9 follow-up studies	DN	T1D	rs7588550	*ERBB4*	1.52	2.10×10^{-7}	1	9.38×10^{-1}	rs11686852	0.92	4.35 × 10-4	*ERBB4*	0.3217
Sandholm *et al.*[10]	Finnish	ESRD	T1D	rs4972593	*SP3 -CDCA7*	1.81	3.85×10^{-8}	0.93	7.62×10^{-2}	rs10930608	0.89	3.13 × 10-4	*LOC100129456*	0.2041
Sandholm *et al.*[11]	Finnish (FinnDiane +7 foolow-up studies)	U-AER	T1D	rs2410601	*PSD3 -SH2D4A*	1.08	3.85×10^{-6}	0.96	3.57×10^{-1}	rs11986186	1.07	8.10 × 10-2	*PSD3*	0.0365
Germain *et al.* [12]	European	DN	T1D	rs1326934	*SORBS1*	1.20'	9.00×10^{-3}	1.04	3.60×10^{-1}	rs149502251	1.54	1.23 × 10-2	*SORBS1*	0.0095

T1D: type 1 diabetes mellitus, T2D: type 2 diabetes mellitus, SNP:Single nucleotide polymorphism, OR: Odds ratio, DN: diabetic nephropathy, U-AER: Urinary Albumin Excretion rate, ESRD: end-stage renal disease

* rs182468383 is not in 1000G reference panel

1. Diabetes 52: 2848-2853, 2003
2. Diabetes 54: 1171-1178, 2005
3. Diabetes 56: 975-983, 2007
4. Diabetes 58: 1403-1410, 2009
5. Kidney Int 80: 105-111, 2011
6. Diabet Med 26: 1090-1098, 2009
7. PLoS Genet 6:e1000842, 2010
8. Kidney Int 79: 563-572, 2011
9. PLoS Genet 8: e1002921, 2012
10. J Am Soc Nephrol 24:1537-1543, 2013
11. Diabetologia 57:1143-1153, 2014
12. Diabetologia 58(3):543-548 2015
