# Supplementary material for: A variant within the FTO confers susceptibility to diabetic nephropathy in Japanese patients with type 2 diabetes
Source: PLoS One. 2018 Dec 19;13(12):e0208654. doi: 10.1371/journal.pone.0208654 (PMC6300288; doi:10.1371/journal.pone.0208654)
Supplement: S5 Table — (PDF) [file pone.0208654.s008.pdf]

S5 Table. Six SNP loci associated with diabetic nephropathy including microalbuminuria (Meta-analysis in Discovery Stage, P < 5 x 10^{-7}) in Japanese patients with type 2 diabetes.

SNP ID *Gene*	Alleles		Study	RAF*		OR(95% CI)	*P*-value	*P*het
	**Effect**	**Non-Effect**		**Case**	**Control**			
rs56094641	G	A	Stage-1, set-1	0.238	0.211	1.17(1.09-1.25)	1.10x10^{-5}	
FTO			Stage-1, set-2	0.233	0.203	1.19(1.04-1.38)	1.35x10^{-2}	
			Stage-2	0.246	0.209	1.23(1.09-1.39)	9.0x10^{-4}	
			**Combined**			**1.18(1.12-1.25)**	**2.43x10^{-9}**	**0.75**
rs78954674	A	G	Stage-1, set-1	0.162	0.143	1.17(1.08-1.28)	2.15x10^{-4}	
			Stage-1, set-2	0.154	0.133	1.22(1.02-1.45)	2.88x10^{-2}	
			Stage-2	0.168	0.137	1.27(0.78-1.47)	1.30x10^{-3}	
			**Combined**			**1.20(1.12-1.28)**	**1.11x10^{-7}**	**0.65**
rs16977473	G	A	Stage-1, set-1	0.941	0.925	1.31(1.16-1.48)	1.04x10^{-5}	
			Stage-1, set-2	0.931	0.93	1.0(0.81-1.30)	8.25x10^{-1}	
			Stage-2	0.939	0.916	1.44(1.18-1.76)	4.00x10^{-4}	
			**Combined**			**1.29(1.18-1.41)**	**1.62x10^{-7}**	**0.09**
rs895157	G	T	Stage-1, set-1	0.127	0.108	1.23(1.12-1.36)	2.60x10^{-5}	
			Stage-1, set-2	0.124	0.112	1.14(0.94-1.37)	1.81x10^{-1}	
			Stage-2	0.135	0.109	1.28(1.09-1.50)	2.30x10^{-3}	
			**Combined**			**1.23(1.14-1.32)**	**1.62x10^{-7}**	**0.62**
rs16940484	T	C	Stage-1, set-1	0.325	0.299	1.15(1.07-1.22)	5.03x10^{-5}	
			Stage-1, set-2	0.324	0.295	1.15(1.01-1.31)	2.99x10^{-2}	
			Stage-2	0.323	0.296	1.14(1.02-1.27)	2.28x10^{-2}	
			**Combined**			**1.14(1.09-1.21)**	**2.44x10^{-7}**	**0.99**
rs73048515	C	T	Stage-1, set-1	0.983	0.974	1.58(1.28-1.96)	2.47x10^{-5}	
			Stage-1, set-2	0.983	0.979	1.21(0.77-1.88)	4.10x10^{-1}	
			Stage-2	0.986	0.975	1.75(1.21-2.56)	3.30x10^{-3}	
			**Combined**			1.56(1.32-1.85)	**4.71x10^{-7}**	0.44

OR: Odds Ratio, 95%CI: 95% confidence interval, RAF: risk allele frequency
