# Supplementary material for: A variant within the FTO confers susceptibility to diabetic nephropathy in Japanese patients with type 2 diabetes
Source: PLoS One. 2018 Dec 19;13(12):e0208654. doi: 10.1371/journal.pone.0208654 (PMC6300288; doi:10.1371/journal.pone.0208654)
Supplement: S6 Table — (PDF) [file pone.0208654.s009.pdf]

S6 Table. Six SNP loci associated with diabetic nephropathy including microalbuminuria (Meta-analysis in Discovery Stage, $P<5x10^{-7}$) in Japanese patients with type 2 diabetes.

**SNP ID** *Gene* **Chromosome**	**Alleles** **Effect**	**Non-Effect**		**OR(95% CI)**	***P*-value**	***P*het**	**RAF***
rs56094641	**G**	**A**	Japanese discovery set	1.18(1.12-1.25)	$2.425x10^{-9}$	0.7535	0.173
FTO			Japanese all replication set	1.11(0.93-1.32)	0.2578		
Ch16			Japanese all combined set	1.18(1.12-1.24)	$1.64x10^{-9}$		
			European replication set	1.04(0.96-1.13)	0.6416		0.434
rs78954674	A	G	Japanese discovery set	1.20(1.12-1.28)	$1.107x10^{-7}$	0.6454	0.149
WWC1			Japanese all replication set	0.95(0.77-1.16)	0.6101		
Ch5			Japanese all combined set	1.17(1.10-1.25)	$1.06x10^{-6}$		
			European replication set	1.01(0.81-1.27)	0.8084		0.046
rs16977473	G	A	Japanese discovery set	1.28(1.18-1.41)	$1.6227x10^{-7}$	0.0887	0.096
CGNL1			Japanese all replication set	1.12(0.85-1.47)	0.4152		
Ch15			Japanese all combined set	1.27(1.15-1.39)	$1.83x10^{-7}$		
			European replication set	1.19(0.81-1.75)	0.7769		0.016
rs895157	G	T	Japanese discovery set	1.23(1.14-1.32)	$1.623x10^{-7}$	0.6195	0.139
PRCD			Japanese all replication set	1.04(0.83-1.30)	0.7476		
Ch17			Japanese all combined set	1.2(1.11-1.29)	$1.17x10^{-6}$		
			European replication set	0.96(0.87-1.08)	0.4431		0.189
rs16940484	T	C	Japanese discovery set	1.14(1.09-1.21)	$2.435x10^{-7}$	0.9865	0.322
TTC39C			Japanese all replication set	1.02(0.87-1.20)	0.7874		
Ch18			Japanese all combined set	1.13(1.08-1.19)	$5.80x10^{-7}$		
			European replication set	1.04(0.82-1.33)	0.3193		0.15
rs73048515	C	T	Japanese discovery set	1.56(1.32-1.85)	$4.709x10^{-7}$	0.4369	0.0014
LOC107986400			Japanese all replication set	1.23(0.78-1.96)	0.3732		
Ch5			Japanese all combined set	1.52(1.28-1.79)	$4.74x10^{-7}$		
			European replication set	1.10(0.94-1.28)	0.2529		0.073

CI, confidence interval; OR, odds ratio; *P*het, *P*-value for Cochran's Q-test for heterogeneity; RAF, risk allele frequency; SNP, single-nucleotide polymorphism.
*Risk allele frequency in each populations.
Data are from 1000 Genomes Project Phase 3 allele frequencies (JPT and EUR)
