# Supplementary material for: A variant within the FTO confers susceptibility to diabetic nephropathy in Japanese patients with type 2 diabetes
Source: PLoS One. 2018 Dec 19;13(12):e0208654. doi: 10.1371/journal.pone.0208654 (PMC6300288; doi:10.1371/journal.pone.0208654)
Supplement: S7 Table — (PDF) [file pone.0208654.s010.pdf]

S7 Table. In silico replication of candidate loci for Diabetic Nephropathy in the data from the SUMMIT Consortium

SNP ID	*GENE*	Effect Allele	beta	se	P_value	OR	95%CI	n (Case and Control)	R^2	RAF*
DN (macroalubuminuria/ESRD)										
rs56094641	*FTO*	G	0.0620974	0.055827	0.2996	1.064066	0.954644-1.186029	3460	0.99699	0.434
rs895157	*PRCD*	C	-0.0856538	0.062368	0.1952	0.917912	0.79567-1.058934	3457	0.946235	0.189
rs10144968	*RAD51B-ZFP36L1*	G	-0.0966486	0.05669	0.2888	0.907875	0.796764-1.034482	3458	0.983975	0.208
rs13306536	*LRP8*	T								0
rs7544082	*TRABD2B*	A	N/A	N/A	N/A	N/A	N/A	N/A	N/A	0.213
rs11101179	*CHAT*	C	N/A	N/A	N/A	N/A	N/A	N/A	N/A	0.312
rs710375	*CCNH-TMEM161B*	T	-0.1111662	0.048236	0.03582	0.89479	0.800247-1.000503	3461	1	0.381
DN(microalbuminuria/macroalbuminuria/ESRD)										
rs56094641	*FTO*	G	0.0422469	0.042581	0.6416	1.043152	0.959693-1.133868	5195	0.99841	0.434
rs78954674	*WWC1*	A	0.0125272	0.104134	0.8084	1.012606	0.808503-1.268232	4898	0.8458125	0.046
rs16977473	*CGNL1*	G	0.1735347	0.194502	0.7769	1.189502	0.808278-1.750529	3042	0.99984	0.016
rs895157	*PRCD*	C	-0.0315846	0.051151	0.4431	0.968909	0.868653-1.080735	5191	0.9458	0.189
rs16940484	*TTC39C*	T	0.0429714	0.116024	0.3193	1.043908	0.816501-1.33465	2239	0.99149	0.15
rs73048515	*LOC107986400*	C	0.0944544	0.080088	0.2529	1.099059	0.942086-1.282186	5192	0.957772	0.073

OR:Odds Ratio, 95%Cl: 95% Confiderence Interval

NA data is not available

*Data are from 1000 Genomes Project Phase 3 allele frequencies (EUR)
