# Supplementary material for: A variant within the FTO confers susceptibility to diabetic nephropathy in Japanese patients with type 2 diabetes
Source: PLoS One. 2018 Dec 19;13(12):e0208654. doi: 10.1371/journal.pone.0208654 (PMC6300288; doi:10.1371/journal.pone.0208654)
Supplement: S8 Table — (PDF) [file pone.0208654.s011.pdf]

Contributors for SUMMIT consortium

Partner	Name	Position
1	Michael Mark	Coordinator, WP6 leader
Boehringer-Ingelheim	Markus Albertini	Project manager
Ingelheim, Germany	Carine Boustany	Chronic Kidney Disease, Head of Lab
	Alexander Ehlgen	Transmed
	Martin Gerl	Biomarker & Bioanlysis, Groupleader
	Jochen Huber	In vivo Scientist CMDR, Head of Lab
	Corinna Schölch	Biomarker & Bioanlysis, Head of Lab
	Heike Zimdahl-Gelling	Pharmacogenomics, Head of Lab
2	Leif Groop	Prof. Endocrinology; Coordinator Managing entity IMI-JU; PI; WP1 and WP6 leader
Lund University	Elisabet Agardh	Prof. Ophthalmology
Clinical Research Centre	Emma Ahlqvist	Postdoc
Malmö, Sweden	Tord Ajanki	Communication strategist
	Nibal Al Maghrabi	Research nurse
	Peter Almgren	Biostatistician
	Jan Apelqvist	Diabetologist
	Eva Bengtsson	Assis. Prof. Cardiovascular research
	Lisa Berglund	Postdoc
	Harry Björckbacka	Assis. Prof. Cardiovascular research
	Ulrika Blom-Nilsson	LUDC administrator
	Mattias Borell	Website, server management
	Agneta Burström	Research nurse
	Corrado Cilio	Assoc. Prof. Cellular autoimmunity
	Magnus Cinthio	Assist. Prof. Electrical Measurements, Lund Technical University
	Karl Dreja	Nephrologist
	Pontus Dunér	Postdoc Exp. Cardiovasc. Research
	Daniel Engelbertsen	PhD student Exp. Cardiovasc. Research
	Joao Fadista	Postdoc
	Maria Gomez	Assoc. Prof. Cardiovascular disease, WP4 co-leader
	Isabel Goncalves	Assis. Prof. Cardiovascular research
	Bo Hedblad	Prof. Cardiovascular epidemiology
	Anna Hultgårdh	Prof. Vessel Wall Biology
	Martin E. Johansson	Pathologist
	Cecilia Kennbäck	Laboratory Engineer
	Jasmina Kravic	Database manager
	Claes Ladenvall	Genetic statistician
	Åke Lernmark	Prof. Type 1 diabetes and celiac disease
	Eero Lindholm	Physician, Researcher Diabetic Complications
	Charlotte Ling	Assist. Prof. Epigenetics
	Holger Luthman	Prof. Medical genetics
	Olle Melander	Assoc. Prof. Hypertension and cardiovascular disease
	Malin Neptin	Biomedical analyst
	Jan Nilsson	Prof. Experimental Cardiovascular research, WP3 leader
	Peter Nilsson	Prof. Internal medicine
	Tobias Nilsson	PhD student Electrical Measurements, Lund Technical University
	Gunilla Nordin Fredriksson	Prof. Cardiovascular research
	Marju Orho-Melander	Prof. Genetic epidemiology
	Emilia Ottoson-Laakso	PhD student
	Annie Persson	Research nurse
	Margaretha Persson	Laboratory Engineer
	Mats-Åke Persson	Database manager

	Jacqueline Postma	Project manager
	Elisabeth Pranter	Research nurse
	Sara Rattik	PhD student Exp. Cardiovasc. Research
	Gunnar Sterner	Chief physician Internal Medicine Research Unit
	Lilian Tindberg	Research nurse
	Maria Wigren	Postdoc Exp. Cardiovasc. Research
	Anna Zetterqvist	PhD student
	Mikael Åkerlund	Postdoc
	Gerd Östling	Laboratory Engineer
3	Timo Kanninen	Technical director; PI
Biocomputing Platforms	Anni Ahonen-Bishopp	Software development manager
(BC Platforms)	Anita Eliasson	Financial and administrative director
Espoo, Finland	Timo Herrala	System (server) specialist
	Päivi Tikka-Kleemola	Service manager
4	Anders Hamsten	Prof. Cardiovascular disease; Atherosclerosis Research Unit; PI
Karolinska Institute	Christer Betsholtz	Prof. Vascular biology
Stockholm, Sweden	Ami Björkholm	Administrator
	Ulf de Faire	Professor emeritus Cardiovascular epidemiology
	Fariba Foroogh	Research engineer
	Guillem Genové	Scientist
	Karl Gertow	Research Assist. Prof. Cardiovascular genetics
	Bruna Gigante	Assoc. Professor Cardiovascular epidemiology
	Bing He	Postdoc
	Karin Leander	Assoc. Professor Cardiovascular epidemiology
	Olga McLeod	Postdoc
	Maria Nastase-Mannila	Postdoc
	Jaako Patrakka	Postdoc
	Angela Silveira	Assoc. Prof. Cardiovascular genetics
	Rona Strawbridge	Postdoc
	Karl Tryggvason	Prof. Medical Chemistry
	Max Vikström	Statistician
	John Öhrvik	Professor
	Anne-May Österholm	Postdoc
5	Barbara Thorand	Nutritional scientist, epidemiologist
Helmholtz Centre	Christian Gieger	Statistician
Munich, Germany	Harald Grallert	Biologist
	Tonia Ludwig	Statistician
	Barbara Nitz	Scientist
	Andrea Schneider	Data manager
	Rui Wang-Sattler	Scientist
	Astrid Zierer	Statistician
6	Giuseppe Remuzzi	Institute director; PI
Mario Negri Institute for	Ariela Benigni	Head of department Molecular Medicine
Pharmacological Research	Roberta Donadelli	Scientist
	Maria Domenica Lesti	Researcher
Bergamo, Italy	Marina Noris	Head Laboratory Immunology and genetics of transplantation and rare diseases
	Norberto Perico	Senior scientist
	Annalisa Perna	Biostatistician
	Rossella Piras	Postdoc
	Piero Ruggenenti	Head of department Renal medicine, Assist. Prof. Nephrology and dialysis

	Erica Rurali	Postdoc
7	David Dunger (att: Jane Horsford)	Prof. Paediatrics; PI
University of Cambridge	Ludo Chassin	Senior Data Manager
UK	Neil Dalton, London	Clinical biochemistry
	John Deanfield, London	Paediatric cardiology
	Jane Horsford	PA to Prof. Dunger
	Clare Rice	Operations manager/financial contact
	James Rudd	Cardiovascular imaging
	Neil Walker	Head Data services
	Karen Whitehead	Technician
	Max Wong	Postdoc
8	Helen Colhoun	Prof. Public health and epidemiology; PI; Vice coordinator Managing entity; WP2 leader
	Fiona Adams	
University of Dundee	Tahira Akbar	PA to Helen Colhoun
Scotland	Jill Belch	Prof. Vasucular disease
	Harshal Deshmukh	PhD student
	Fiona Dove	
	Angela Ellingford	NHS Tayside Diabetic Retinopathy Screening Programme manager
	Bassam Farran	Statistician
	Mike Ferguson	Dean of research Biological chemistry and drug discovery
	Gary Henderson	
	Graeme Houston	Consultant radiologist/senior lecturer
	Faisel Khan	Reader, Vascular & Inflammatory Diseases Research Unit
	Graham Leese	Consultant diabetologist/reader
	Yiyuan Liu	PhD student
	Shona Livingstone	Senior statistician
	Helen Looker	Epidemiologist
	Margaret McCann	Project assistant
	Stuart McGurnaghan	Lead data programmer
	Andrew Morris	Prof. Diabetic medicine
	David Newton	
	Colin Palmer	Prof. Pharmacogenomics
	Ewan Pearson	Consultant diabetologist/senior lecturer
	Gillian Reekie	Research Nurse
	Natalie Smith	Research Nurse
9	Angela Shore	Prof. Cardiovascular Science, PI
Peninsula Medical School	Kuni Aizawa	Postdoc
Exeter, UK	Claire Ball	Research nurse
	Nick Bellenger	Cardiologist
	Francesco Casanova	Associate Research Fellow Vascular medicine
	Tim Frayling	Prof. Genetics
	Phil Gates	Senior lecturer Cardiovascular science
	Kim Gooding	Postdoc Vascular medicine
	Andrew Hatttersley	Prof. Molecular medicine
	Roland Ling	Consultant opthalmologist
	David Mawson	Research technician
	Robin Shandas	Prof. Bioengineering (Colorado)
	David Strain	Stroke physician, clinical lecturer
	Clare Thorn	Postdoc Vascular medicine

10	**Ulf Smith**	Prof. ; PI
University of Gothenburg	Ann Hammarstedt	Researcher Molecular and clinical medicine
Sweden	Hans Häring	Prof. University of Tübingen
	Oluf Pedersen	Prof. Steno Centre, Copenhagen
	Georgio Sesti	Prof. Universtiy of Catanzaro
11	**Per-Henrik Groop**	Prof. Diabetes genetics; PI
	Emma Fagerholm	PhD student, genetics
Folkhälsan	Carol Forsblom	Clinical coordinator
Helsinki, Finland	Valma Harjutsalo	
	Maikki Parkkonen	Laboratory manager
	Niina Sandholm	DSc(PhD); GWAS and bioinformatics
	Nina Tolonen	MD PhD
	Iiro Toppila	BSc, bioinformatician
	Erkka Valo	MSc, bioinformatician
12	**Veikko Salomaa**	Prof. Epidemiology; PI; **deputy leader WP2**
The National Institute for Health and Welfare	Aki Havulinna	DSc. (tech), statistician
Helsinki, Finland	Kati Kristiansson	Postdoc
	Pia Okamo	THL press officer
	Tomi Peltola	
	Markus Perola	Professor
	Arto Pietilä	Statistician
	Samuli Ripatti	Professor, Statistics
	Marketta Taimi	Research assistant
13	**Seppo Ylä-Herttuala**	Prof.; PI; **WP4 leader**
University of Eastern Finland	Mohan Babu	PhD student
Kuopio, Finland	Marike Dijkstra	PhD student
	Erika Gurzeler	PhD student
	Jenni Huusko	PhD student
	Ivana Kholová	Postdoc
	Markku Laakso	Prof.
	Mari Merentie	PhD student
	Marja Poikolainen	PA Prof Ylä-Herttuala
14	**Mark McCarthy**	Prof. Human type 2 diabetes; Oxford Centre for Diabetes, Endocrinology and Metabolism; Wellcome Trust Centre for Human Genetics; PI; **deputy leader WP1**
University of Oxford	Chris Groves	Technical staff
UK	Thorhildur Juliusdottir	PhD student
	Fredrik Karpe	PI OCDEM
	Vasiliki Lagou	Postdoc
	Andrew Morris	Wellcome Trust Senior Fellow; Bioinformatics and statistical genetics
	Will Rayner	Database manager
	Neil Robertson	Informatics
	Natalie van Zuydam	Postdoc
15	**Claudio Cobelli**	Prof. ; PI; **WP5 leader**
University of Padova	Barbara Di Camillo	Assist. Prof.
Italy	Francesca Finotello	PhD student
	Francesco Sambo	Postdoctoral fellow
	Gianna Toffolo	Prof.
	Emanuele Trifoglio	PhD student

16	**Riccardo Bellazzi**	Prof. Bioengineering; PI; **deputy leader WP5**
	Nicola Barbarini	Postdoctoral fellow
University of Pavia	Mauro Bucalo	Software engineer
Italy	Christiana Larizza	Assist. Prof.
	Paolo Magni	Assoc. Prof.
	Alberto Malovini	Postdoctoral fellow
	Simone Marini	Postdoctoral fellow
	Francesca Mulas	Postdoctoral fellow
	Silvana Quaglini	Prof.
	Lucia Sacchi	Assist. Prof.
	Francesca Vitali	
17	**Ele Ferrannini**	Prof. Medicine; PI
	Beatrice Boldrini	Postdoctoral fellow
University of Pisa	Michaela Kozakova	Senior investigator Medical Pathophysiology
Italy	Andrea Mari	Senior researcher Biomedical engineering (ISIB-CNR, Padova)
	Carmela Morizzo	Biologist, Sonographer Cardiovascular ultrasound
	Lucrecia Mota	EGIR administrative office
	Andrea Natali	Assoc. Prof. Medicine
	Carlo Palombo	Assoc. Prof. Medicine; **deputy leader WP3**
	Elena Venturi	Researcher
	Mark Walker	Prof. Molecular diabetic medicine (Univ Newcastle-upon-Tyne)
18	**Carlo Patrono**	Prof.Pharmacology; PI
Catholic University of Rome	Francesca Pagliaccia	PhD student
Italy	Bianca Rocca	Assist. Prof. Pharmacology
19	**Pirjo Nuutila**	Prof. ; PI
University of Turku	Johanna Haukkala	PhD student
Finland	Juhani Knuuti	Prof. ; Director Turku PET Centre
	Anne Roivainen	Prof.
	Antti Saraste	Adj. Prof.
20	**Paul McKeague**	Prof. Genetic Epidemiology; PI
University of Edinburgh	Norma Brown	Research administrator, Public Health Services
Scotland	Marco Colombo	Bioinformaticist
21	**Birgit Steckel-Hamann**	Deputy coordinator; PI, Manager IMI, LRL
Eli Lilly	Krister Bokvist	Biostatistician
	Sudha Shankar	Diabetologist
	Melissa Thomas	Translational Science
22	**Li-ming Gan**	Prof.; Translational Science Director Cardiovascular Disease; PI, **WP3 leader**
AstraZeneca	Suvi Heinonen	PhD, Internal AZ postdoc, Bioscience
	Ann-Cathrine Jönsson-Ryl	PhD, Assoc. Prof., Team Leader Bioscience, **WP4 leader**
	Remi Momo	Postdoctoral fellow
	Volker Schnecke	Informatician Translational Science, **WP5 leader**
	Robert Unwin	Translational Science Director Diabetic Nephropathy
	Anna Walentinsson	Geneticist Translational Science
	Carl Whatling	Bioscientist
23	**Everson Nogoceke**	Pre-clinical and clinical aspects of metabolic and vascular disease; PI; **WP2 leader**
Roche	Gonzalo Durán Pacheco	Senior Research Statistician

	Ivan Formentini	Biomarker & Experimental Medicine Leader
	Thomas Schindler	Pre-clinical and clinical and clinical biomarkers
24	**Piero Tortoli**	Professor of Electronics
University of Florence	Luca Bassi	Postdoctoral fellow
	Enrico Boni	Postdoctoral fellow
	Alessandro Dallai	Postdoctoral fellow
	Francesco Guidi	Technician
	Matteo Lenge	PhD student
	Riccardo Matera	PhD student
	Alessandro Ramalli	PhD student
	Stefano Ricci	Assist. Prof.
	Jacopo Viti	PhD student
25	**Bernd Jablonka**	SAD internal IMI coordinator
Sanofi-aventis	Dan Crowther	Biomarker researcher
	Johan Gassenhuber	Biostatistician
	Sibylle Hess	Biomarker researcher
	Thomas Hübschle	Pharmacologist Diabetes
	Hans-Paul Juretschke	Imaging
	Hartmut Rütten	Head Translational Medicine
	Thorsten Sadowski	Pharmacologist Diabetes
	Paulus Wohlfart	Pharmacologist Diabetes
26	**Julia Brosnan**	Biochemist, (pre)clinical research CVD, Pfizer US; **WP2 leader**
Pfizer	Valerie Clerin	Cardio-renal biologist, WP2
	Eric Fauman	Computational biologist
	Craig Hyde	Statistician
	Anders Malarstig	Human genetics, Pfizer Europé; **WP1 leader**
	Nick Pullen	Renal Disease Research Director
	Mera Tilley	
	Theresa Tuthill	Imaging specialist
	Ciara Vangjeli	Cardiovascular genetic epidemiologist, Pfizer Europe
	Daniel Ziemek	Computational biologist
